# Coral Genetic Structure in the Western Indian Ocean Mirrors Ocean Circulation and Thermal Stress History

**DOI:** 10.1111/eva.70206

**Published:** 2026-02-17

**Authors:** Annie S. Guillaume, Stéphane Joost, Sarvanen Curpen, Danishta Dumur Neelayya, Luxmibye Harree‐Somah, Oocheetsing Sadasing, Luca Saponari, Charlotte Dale, Léo Barret, Nina Andrews, Sanjeev Kumar Leckraz, Ronnie François, Vasisht Seetapah, Vinayaganidhi Munusami, Suraj Bacha Gian, Reshad Jhangeer‐Khan, Terence Mahoune, Pramod Kumar Chumun, Manuel Poretti, Véronique Berteaux‐Lecellier, Gael Lecellier, Oliver Selmoni

**Affiliations:** ^1^ Geospatial Molecular Epidemiology Group (GEOME), Laboratory for Biological Geochemistry (LGB), École Polytechnique Fédérale de Lausanne (EPFL) Lausanne Switzerland; ^2^ Mauritius Oceanography Institute Albion Mauritius; ^3^ Nature Seychelles Roche Caiman Seychelles; ^4^ James Cook University Townsville City Queensland Australia; ^5^ Bee Ecological Consulting Mahé Seychelles; ^6^ Marine Conservation Society Seychelles Beau Vallon Seychelles; ^7^ Albion Fisheries Research Centre Albion Mauritius; ^8^ Eco‐Sud Blue Bay Mauritius; ^9^ Reef Conservation Mauritius; ^10^ Shoals Rodrigues Rodrigues Mauritius; ^11^ Seychelles Parks and Gardens Authority Seychelles; ^12^ United Nations Development Programme Mauritius and Seychelles Port Louis Mauritius; ^13^ School of BioSciences University of Melbourne Melbourne Victoria Australia; ^14^ Innovation, applications et transfert pour la Santé des Ecosystèmes tropicaux EMR9001 SantEco (CNRS‐IRD‐UNC‐UR) UMR250 ENTROPIE Noumea New Caledonia; ^15^ Institut des Sciences Exactes et Appliquées (ISEA) EA7484‐UNC Nouméa New Caledonia; ^16^ Department of Embryology Carnegie Institution for Science Baltimore Maryland USA; ^17^ Department of Plant Biology Carnegie Institution for Science Stanford California USA; ^18^ Department of Geography University of Zurich Zurich Switzerland

**Keywords:** adaptive potential, conservation management, genotype–environment associations, reef connectivity, seascape genomics, thermal adaptation

## Abstract

Global warming and rising sea temperatures are pushing many reef‐building coral species towards extinction. As thermal tolerance in corals is partially heritable, identifying genes under thermal selection is critical for targeted biodiversity management. However, it remains unclear how large connectivity breaks (> 100 km of open sea) might affect the spread of adaptive alleles for different coral species in discontinuous reef networks such as the Western Indian Ocean (WIO). To address this, we applied a seascape genomics approach to model (i) population structure and (ii) thermal adaptive potentials for two keystone coral species, *Acropora muricata* and *Pocillopora damicornis* , across the WIO. Northern reefs in the Seychelles were largely genetically isolated from southern reefs in Rodrigues and Mauritius for both species, potentially driven by regional oceanographic barriers. Isolation‐by‐resistance calculated from ocean currents during reproductive months better explained regional genetic differences than isolation‐by‐distance alone. Spatial patterns of genetic variation were best captured by variables representing thermal stress, including sea surface temperature variability, accumulating heat stress, and fine‐scale reef structure. Using these variables in genotype–environment association (GEA) analyses identified hundreds of loci under putative thermal selection, including several linked to genes involved in heat stress responses. We detected 12 molecular functions enriched in *A. muricata* and 20 enriched in *P. damicornis* , generally pertaining to cellular signalling, transport mechanisms, metabolism, and protein quality control, including six genes annotated as the heat‐shock chaperone protein Sacsin for *A. muricata* . We produce species‐specific maps estimating the putative thermally adaptive seascape across the WIO, which, when combined with population structure and previous ocean current models, indicate that the spread of heat adapted genotypes may be inhibited across the WIO. This research provides valuable insights into WIO coral population structure and thermal adaptive potentials to inform local and regional conservation management across the region.

## 1 Introduction

Covering approximately 0.1% of the ocean floor, coral reefs are biodiversity hotspots hosting approximately a quarter of all marine species (Reaka‐Kudla 1997; Spalding and Grenfell 1997). These dynamic ecosystems provide valuable habitat for a variety of taxa, shaping food chains and biogeochemical cycles while also providing important ecosystem services for humans, including food, medicine, coastal protection and tourism (Costanza et al. 2014; Hughes, Barnes, et al. 2017; Moberg and Folke 1999; Roff et al. 2016). The foundation of these ecosystems are reef‐building scleractinian corals, whose calcareous skeletons construct the reef's physical structure (Hughes, Barnes, et al. 2017; Moberg and Folke 1999). Coral reefs are under threat of intensifying anthropogenic‐induced stressors, particularly from global warming and elevated sea temperatures (Bozec et al. 2025; Hughes, Kerry, et al. 2017; Hughes et al. 2020; Ortiz et al. 2021; Otto 2018; Virgen‐Urcelay and Donner 2023). Under stressful conditions, corals can lose their symbiotic algae in a reversible process known as coral bleaching (Helgoe et al. 2024; Hoegh‐Guldberg et al. 2007). As corals rely on their symbionts for metabolic inputs, persistent bleaching can result in widespread coral death (Sully et al. 2022). An estimated 14% of the world's coral cover has been lost from 2009 to 2018 (Souter et al. 2021), and with bleaching‐induced coral mortality on the rise (Bozec et al. 2025; Hughes, Kerry, et al. 2017; Hughes et al. 2020; Virgen‐Urcelay and Donner 2023) there is concern for the impact of climate change on coral reef structures, biodiversity, functioning and productivity globally (Graham et al. 2015; Hughes et al. 2019; Morais et al. 2022; Sully et al. 2022).

Despite the increased frequency and intensity of mass bleaching events, corals appear to adapt to repeated exposure to elevated temperatures (e.g., Bozec et al. 2025; Drury and Lirman 2021; Louis et al. 2016; Palumbi et al. 2014; Schoepf et al. 2015). For example, increased thermal tolerance of coral communities was observed around Palau in the Pacific Ocean in recent years, following multiple acute bleaching events in the last decades (Bruno et al. 2001; Lachs et al. 2023). While these trends could be attributed to acclimatisation, shifts in community structure, or changes in holobiont associations (Gouezo et al. 2019), contemporary within‐population variation in coral heat tolerance suggests some underlying genetic adaptation to elevated temperatures (Lachs et al. 2023; Mumby and Van Woesik 2014). Using a variety of experimental methods (summarised in Selmoni et al. 2024), studies have shown that within‐species thermal variation is heritable, with evidence arising from the Pacific Ocean (e.g., Bay and Palumbi 2014; Selmoni, Rochat, et al. 2020; Selmoni et al. 2021), the Great Barrier Reef of eastern Australia (e.g., Cooke et al. 2020; Dixon et al. 2015; Elder et al. 2022; Fuller et al. 2020; Jin et al. 2016; Lundgren et al. 2013; Quigley et al. 2020), the East Indian Ocean along the west Australian coast (e.g., Thomas et al. 2017, 2022), the Persian Gulf in the Middle East (e.g., Howells et al. 2016, 2021; Kirk et al. 2018; Smith et al. 2022), and the Caribbean Sea (e.g., Drury and Lirman 2021; Dziedzic et al. 2019). As the heritability of thermal variation implies the existence of alleles promoting tolerance to anticipated thermal regimes, identifying candidate genes and populations adapted to historical thermal stresses can provide valuable information to improve spatial mapping of reef vulnerability and inform reef restoration efforts (Selmoni et al. 2024).

Seascape genomics provides a promising framework for disentangling neutral from adaptive processes in in‐situ populations distributed across diverse geographic and environmental ranges (Riginos et al. 2016; Selmoni, Rochat, et al. 2020; Selmoni et al. 2021). Characterising neutral population structure across large spatial extents can reveal how geographic distance and sea currents affect the dispersal of adaptive alleles within and between coral populations (De Mita et al. 2013; Lotterhos and Whitlock 2015; Matz et al. 2020; Selkoe et al. 2016; Selmoni, Rochat, et al. 2020; Weersing and Toonen 2009). Investigating the relationship between genetic distance and geographic or sea distance can provide insights into the mechanisms driving population structure under Isolation‐by‐Distance (IBD; Polato et al. 2010; Wright 1943) or Isolation‐by‐Resistance (IBR; McRae 2006; Thomas et al. 2015), respectively. Seascape genomics also provides a framework for correlating whole genome variation with environmental factors using genotype–environment associations (GEA; Rellstab et al. 2015). Significant associations can indicate candidate loci under selection, and, when reference genomes are available, allow identification of markers near or within genes of interest to infer molecular functions under selection (Storfer et al. 2018). These investigations are particularly important across reef networks for sustaining the long‐term adaptive capacity of coral populations, as simulations indicate that maintaining a reef's coral cover will depend on the immigration of thermally adapted recruits from hotter reefs experiencing temperatures 0.5°C above the local average (Matz et al. 2020). By understanding regional‐scale population structure and identifying reefs hosting putatively adaptive individuals, informed management plans can be developed to sustain long‐term adaptive capacities of coral populations (reviewed in Selmoni et al. 2024).

While strides are being made to understand thermal adaptations and heritability in corals globally, three research gaps remain. First, research is predominantly concentrated around reef systems in the Indo‐Pacific region, notably the Great Barrier Reef in Australia (summarised in Selmoni et al. 2024), where early GEA methods successfully detected candidate genes under thermal selection (Lundgren et al. 2013). Yet, large stretches of coral reefs remain understudied (Shlesinger and van Woesik 2023), including across the Western Indian Ocean (WIO; Carr et al. 2025). The WIO hosts approximately 5% of global coral reefs, which are affected by recurrent short‐ and long‐term bleaching events (Shlesinger and van Woesik 2023; van Woesik and Kratochwill 2022). With most WIO ecosystems labelled as vulnerable (e.g., Seychelles) to critically endangered (e.g., Mauritius Islands) due to human activities and global warming (Obura et al. 2022), more research is needed for informed biodiversity management in this region.

The second knowledge gap is that coral research typically focuses on contiguous reef systems or local isolated reefs (Selmoni et al. 2024), with little investigation of dispersed networks with > 100 km of open sea between reefs. Understanding the impact of dispersed networks on coral connectivity is important, particularly with regards to reef‐building corals with differing reproductive modes (reviewed in Harrison 2011). Predominantly broadcast spawning species tend to show weaker population structuring across contiguous reefs, whereas brooders and clonally reproducing taxa generally have shorter dispersal distances resulting in stronger local structuring (Meziere et al. 2025). Large breaks in reef connectivity likely result in weak local and strong regional population structuring, regardless of reproductive mode, but this hypothesis is yet to be explicitly investigated. Emerging patterns for WIO corals show high genetic connectivity within reef systems, such as the Mascarene (Gélin et al. 2018; Oury et al. 2024) or Seychelles Islands (Burt et al. 2024), with reduced connectivity between reefs over larger oceanic expanses (> 400 km) of the southern WIO. To our knowledge, population connectivity between southern and northern WIO reefs has yet to be evaluated, where population structure analyses can provide a first step towards understanding gene flow across WIO latitudes.

The last gap is that research into coral thermal adaptation is typically conducted on one species at a time (Selmoni et al. 2024; but see Selmoni et al. 2021). In addition to differences in reproductive modes, diversity of morphologies and habitat preferences results in differential responses of corals to selection pressures and stressful events (Guest et al. 2012; Johnston et al. 2022; Yuen et al. 2023). For example, corals with branched morphologies are generally more sensitive to heat stress than those with massive morphologies (Loya et al. 2001). With species‐specific evolutionary trajectories, spatial patterns of population structure and adaptation must be considered across multiple species (Voolstra et al. 2023).

We address these gaps by investigating population structure and thermal adaptation for two coral species sampled across the large, non‐contiguous reef network of the WIO. Specifically, we investigate (i) the population structure of two keystone coral species with contrasting reproductive strategies, and (ii) their molecular adaptations to thermal conditions. To this end, we applied a seascape genomics framework to detect coral reefs in the WIO that putatively host thermal stress‐adapted coral genotypes, to ultimately support local stakeholders develop biodiversity management plans across the WIO (Donovan et al. 2021; Goetze et al. 2021; Pittman et al. 2021; Stefanoudis et al. 2023).

## 2 Materials and Methods

**FIGURE 1:**
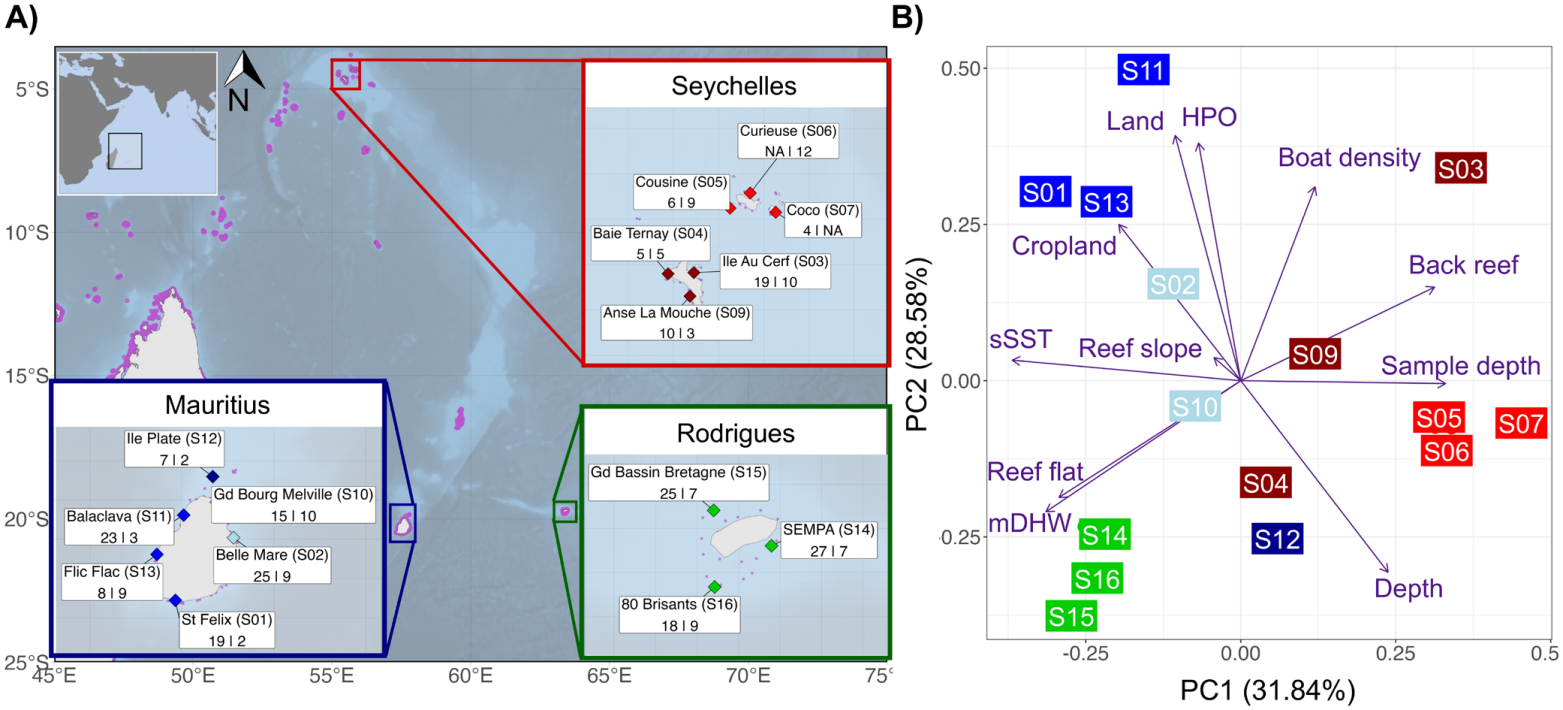
(A) Map of the study area in the Western Indian Ocean (WIO; map insert, top left) indicating the 15 sampling sites distributed around the Seychelles (Mahé: dark red, Praslin: red), and two of the Mascarene Islands of Mauritius (blue) and Rodrigues (green). Names and site identifiers (S#) used throughout this study are provided for each reef in the three regional inserts. The number of genotyped individuals retained after filtering are indicated at each sampling site per species, with values first for *A. muricata* then *P. damicornis* . (B) Environmental characterisation of each study reef (points as labelled text boxes) was undertaken using 10 uncorrelated environmental variables (arrows; Table 1). Variable abbreviations are as follows: HPO = human population density; mDHW = mean degree heating week; sSST = standard deviation of sea surface temperature. Note that ‘Sample depth’ refers to the average sample depth at the study site, while ‘Depth’ refers to depth values extracted from the Allen Coral Atlas, processed at a 250 m radius around the sample coordinates. Reef labels are colour‐coded according to the sub‐region that corresponds to those used in the regional inserts of panel (A).

We focus on two coral species, *Acropora muricata* and *Pocillopora damicornis* , sampled around the northern reefs of the Seychelles, and in southern reefs around Mauritius and Rodrigues (Figure 1a). We first characterised the seascape conditions around each island using open‐access satellite and geomorphic data, from which 15 reefs with contrasting environmental conditions were selected for coral sampling and genotyping. Our framework follows a seascape genomics approach (Selmoni, Rochat, et al. 2020) to (i) map population distributions across the WIO region and (ii) identify putatively adaptive coral genotypes using genotype–environment association (GEA) analyses (Figure S1). We couple these analyses to infer the spatial distribution of WIO reefs that potentially carry genotypes underpinning local adaptation to historic thermal stress for informed reef management planning. All analyses were performed in the R environment (R Core Team 2024), with scripts available on Dryad (Guillaume et al. 2026).

### 2.1 Study Species

*A. muricata* and *P. damicornis* were selected here as two common reef‐building corals of the WIO that represent keystone species (Bridge et al. 2023; Cunning et al. 2018). Evidence from closely related species suggests that thermal tolerance is highly variable within populations (Carr et al. 2025; Humanes et al. 2022; Lachs et al. 2023), driving hypotheses that signatures of local adaptation to thermal stress might be detected in these study species. With distinct life history strategies, independent investigations of population structure and thermal tolerance are warranted. *A. muricata* is predominately a broadcast spawning coral, releasing gametes into the water column in synchronised events during the Austral summer (November to January) when sea temperatures increase (Wijayanti et al. 2019). Larvae are generally competent to settle within 10–14 days of fertilisation (Denis et al. 2014). *A. muricata* is also known to reproduce asexually by fragmentation. In contrast, the *P. damicornis* species complex has mixed reproduction mode across its range (Gélin et al. 2018). Individuals can reproduce sexually via year‐round broadcast spawning following a full moon (Ward 1992) or by producing brooding planula larvae with rapid settlement rates (Gélin et al. 2018). Clonal propagation is also common in this species complex, with the production of asexual larvae or polyps that can disperse over large distances (> 50 km) by free swimming or fixing to rafting objects (Gélin et al. 2017, 2018; Oury et al. 2019; Schmidt‐Roach et al. 2012; Smith et al. 2019).

### 2.2 Sampling Strategy

Fifteen reefs were selected for sampling across three regions of the WIO: six sites around Mauritius, six sites around the Seychelles (three each at Mahé and Praslin) and three sites around Rodrigues (Figure 1a; Table S1). Reefs were selected following the methods of Selmoni, Vajana, et al. (2020b) to maximise environmental contrast for improved power in seascape genomic analyses, while simultaneously considering logistical constraints in collaboration with local stakeholders, including site accessibility and weather conditions during sampling. Full details are provided in the Supporting Information (Supplementary Methods: Selection of sample sites).

The field sampling campaign took place with the assistance of scientists and technical staff of the Mauritius Oceanography Institute (MOI) in January and February 2022 for Mauritius and Seychelles, and in May 2022 for Rodrigues. This sampling campaign was performed within the framework of the Coral Reef Restoration Project of the United Nations Development Programme Mauritius (PIMS 5736, sampling permit in Rodrigues SPA 30) and the Government of Seychelles (sampling permit A0157). Colonies identified as *A. muricata* and *P. damicornis* were sampled for genotyping (up to 30 colonies per site per species) within a radius of 250 m from the coordinates of the sample site. Sampling was restricted to shallow depths (1–4 m) to avoid confounding effects of depth with genetic structuring (e.g., cryptic speciation can be driven by depth; Prata et al. 2022; Rippe et al. 2021), except for three Praslin (SEY) sites with sample depths between 3 and 10 m. Each sample consisted of a 2 cm branch collected with pliers and bagged underwater before being transferred to 80% ethanol and stored at −20°C. A total of 345 *A. muricata* and 403 *P. damicornis* colonies were sampled for DNA extraction and single nucleotide polymorphism (SNP) genotyping (Table S1).

### 2.3 Environmental Characterisation

Seascapes of the 15 study reefs were characterised using 17 environmental and geomorphic variables hypothesised to exert selection pressures on corals (Table S2). These variables were accessed from two publicly available online archives. Environmental variables pertaining to climatic conditions and proximity to anthropogenic activities covering the whole study extent were extracted from the Reef Environment Centralized Information System (RECIFS; Selmoni et al. 2023). These variables were accessed at 5 km^2^ resolutions and described as month‐by‐month environmental variation covering a temporal range of a minimum of 20 years up to December 2021 (i.e., when sampling took place; full details in Table S2). Temporal mean and standard deviations were calculated for degree heating week (DHW; Skirving et al. 2020), sea surface temperature (SST; Skirving et al. 2020), chlorophyll (CHL) and suspended particulate matter (SPM), where CHL and SPM were obtained from the ‘OCEANCOLOUR_GLO_BGC_L4_MY_009_104’ dataset (accessed on 03‐04‐2024; CMEMS 2024). We also retrieved a geomorphic characterisation of the WIO reefs from the Allen Coral Atlas (ACA; Allen Coral Atlas 2022). Available at 10 m^2^ resolution, reef geomorphology was processed as the proportion of reef slope, reef flat, back reef and reef plateau in the 250 m radius around each sample site coordinate. A Spearman's pairwise non‐parametric rank correlation threshold of *ρ* < |0.8| was applied to retain 10 uncorrelated variables for downstream analyses. These were: DEP = depth, mDHW = mean DHW, sSST = standard deviation of SST, CROP = cropland density, LAND = land density, HPOP = human population density, BOAT = boat detection, ReefSlope = proportion of reef slope, ReefFlat = proportion of reef flat, BackReef = proportion of back reef. A principal component analysis (PCA) on these 10 scaled and centred uncorrelated variables was used to characterise the sample sites (Figure 1b).

### 2.4 DNA Extraction and SNP Genotyping

DNA extraction and SNP genotyping followed methods of Selmoni et al. (2021). Briefly, DNA was extracted using a DNeasy Blood and Tissue 96 kit (Qiagen) following the manufacturer's instructions. DNA samples were sent to Diversity Arrays Technology (Canberra, Australia) for quality check screening and genotype‐by‐sequencing using the DArT‐sequencing method (DArT‐seq). DArT‐seq is a restriction site associated DNA sequencing method (RAD‐seq) that combines DArT complexity reduction methods and Illumina NovaSeq technologies to return reduced representation genome sequencing (Jaccoud et al. 2001). Here, we used the restriction enzymes *Pst*I and *Hpa*II (Gawroński et al. 2016; Kilian et al. 2012; Selmoni et al. 2021). Sequence read processing was performed by Diversity Arrays Technology following proprietary DArT analytical pipelines (Sansaloni et al. 2011). This returned bi‐allelic SNPS at a minimum sequence depth of 5 for each species, with a median read coverage of 45.7 (IQR 16.5–117.8) for *A. muricata* and 34.0 (IQR 13.1–104.0) for *P. damicornis* . The relationship between depth of coverage and heterozygosity rates were weak after filtering for missingness at 80% and minor allele frequency (MAF) < 0.5 for both species (*A. muricata: r* ^2^ = 0.06, *t*_8605_ = 5.84, *p* < 0.001; *P. damicornis: r* ^2^ = 0.10, *t*_16098_ = 13.35, *p* < 0.001). To minimise technical bias and potential batch effects, targeted *A. muricata* and *P. damicornis* samples were kept separated and randomly distributed across the respective batches (e.g., 96‐well plates, sequencing lanes) throughout the workflow (DNA purification, library preparation and sequencing). Raw sequence data are available on NCBI BioProject database under accession number PRJNA1277000.

### 2.5 SNP Filtering

The raw DArT‐seq loci were aligned to each species' reference genomes using BLAST, retaining only the SNPs associated with the coral hosts (filtering thresholds: > 70% percentage identity, > 80% overlap identity, > 50 bitscore). The reference genome used for *A. muricata* was the chromosome‐level assembly of *Acropora millepora* (v2.1; GCF_013753865.1; Fuller et al. 2020), and the reference genome for *P. damicornis* was the scaffold‐level assembly of *P. damicornis* (v1; GCF_003704095.1; Cunning et al. 2018).

SNP filtering was performed for each species separately using functions in the *dartR* R package (v. 2.9.7; Gruber et al. 2018; Mijangos et al. 2022). We first removed putatively cryptic individuals likely present for our sampled species (Gélin et al. 2017, 2018; Johnston et al. 2017; Oury et al. 2024), where ‘cryptic’ is defined as genetically distinct groups among sets of colonies identified in situ as the same species (Grupstra et al. 2024; Riginos et al. 2024). To this end, we performed soft filtering for loci and individual missingness (50% threshold each), then ran a principal coordinate analysis (PCoA) on Euclidean distances of the genotype matrix (an ordination‐based method that can handle missing data). We used PCoA axes to visually identify clusters of genetically distinct individuals co‐occurring with most of the sampled individuals. Individuals from these clusters are putatively cryptic and were removed, as recommended by standard guidelines (Grupstra et al. 2024; Riginos et al. 2024). We then identified and removed clonal genotypes on a hard filtered dataset (loci and individuals each pruned for 80% missingness). Clones were identified as those sharing over 95% or 90% of their genotypes for *A. muricata* and *P. damicornis* , respectively, using the *gl.report.replicates* function in the *dartR.base* package (v. 1.0.5; Mijangos et al. 2022). The choice of threshold corresponds to the separation between first‐degree relatives and replicated individuals in the histogram of pairwise relatedness values per species. For each group of putative clones, we retained the one individual with the least missing SNP data when genetic similarity was found. We identified and removed another cluster of putatively cryptic individuals for *P. damicornis* as per the first cryptic filtering step. Finally, we performed hard filtering on the genotype matrix pruned for cryptic and clonal individuals, applying a missingness threshold of 80% for loci and individuals, before excluding rare alleles with a MAF < 5%. Global MAF thresholds were applied to retain population‐specific SNP variants for downstream population structure and genotype–environment analyses.

We genetically confirmed the species identity of retained *Pocillopora* samples as *P. damicornis* using DArT‐seq within the ITS‐2 and 18S ribosomal DNA regions (Table S3). DArT‐seq reads were first mapped against available rDNA from different *Pocillopora* species and related outgroup species (*Seriatopora* spp. and *Stylophora pistillata* ), using minimap2 (v2.26) with parameters “‐ax sr”. Then, nucleotide sequences from the longest ITS‐2 and 18S regions present within all sequenced samples were aligned against the available rDNA sequences using both BLASTn (v2.14.0; standard parameters) and clustalW (v1.2.4; standard parameters). ClustalX was finally used to visualise clustalW alignments. BLASTn results and clustalW alignments, alongside genbank accession numbers used for rDNA sequences, are summarised in Table S3.

Last, we assessed linkage disequilibrium (LD) amongst SNPs using functions from *dartR* to create an LD map of the genome, with a maximum pairwise limit of 15 kbp (the distance of estimated LD decay to *r* ^2^ < 0.05, based on whole genome sequence data of *A. millepora*; Fuller et al. 2020) before pruning SNPs with an LD > 0.2. As expected when using RADseq data, we found minimal effect of LD pruning on population structure (assessed using a PCoA), nor evidence of SNPs clumping in a PCA (assessed using *pcadapt*; Luu et al. 2017; Privé et al. 2020). We therefore retained all loci for downstream analyses.

### 2.6 Neutral Genetic Structure

Neutral genetic structure was assessed for each species using complementary methods: (i) Principal Coordinate Analysis (PCoA) and (ii) Sparse Nonnegative Matrix Factorization (sNMF). We first produced a putatively neutral genomic dataset (hereafter termed ‘neutral genotype matrix’) by removing outlier loci identified using a genome scan of the filtered genotype matrix with *pcadapt*. Loci were attributed *p*‐values based on their Mahalanobis distances along the two retained PCA axes of the genotype matrix. *p*‐values were corrected for multiple testing using the *qvalue* R package (2.36.0; Storey et al. 2024) to calculate false discovery rates (FDR; Storey and Tibshirani 2003), with outlier loci having a *q* < 0.05.

Contemporary population structure was assessed using a Gower PCoA clustering mixture model in *dartR*, constructed from a distance matrix of allele frequency differences among individuals calculated from the neutral genotype matrix. We retained 2 PCo axes for both species before allocating individuals to genetic groups using a hierarchical cluster analysis on the retained scaled axes. Ancestral population structure was assessed using sNMF algorithms with the *LEA* R package (v3.16.0; Frichot and François 2015). Using a cross‐entropy validation for *K* = 1–10 over five repetitions with an alpha = 100, we identified the number of ancestral populations to retain, using the *K* values resulting in the lowest average cross entropy to compute the sNMF. Membership assignment of each individual to the *K* ancestral populations was represented as proportions in a barplot. We calculated pairwise genetic differences between the sample sites using *F*_ST_ (Wright 1969, 1978) with functions in *dartR*.

### 2.7 Isolation‐By‐Distance Versus Isolation‐By‐Resistance Analyses

Patterns of Isolation‐by‐Distance (IBD) and Isolation‐by‐Resistance (IBR) were identified between reefs by regressing linearised genetic distance (i.e., *F*_ST_/1 − *F*_ST_; Rousset 1997) against Euclidean or sea distances, respectively. IBD was calculated from the log_10_ of Euclidean distances obtained by converting degree latitude and longitude to cartesian coordinates with the *sph2car* function (*sphereplot* R package v1.5.1; Robotham 2022). IBR was calculated from the log_10_ of sea distances derived from ocean current data, following methods in Selmoni, Rochat, et al. (2020). Briefly, monthly and annual sea distances were obtained from rasters describing average direction and strength of surface currents across the WIO. These publicly available rasters were downloaded via RECIFS (Selmoni et al. 2023) at a spatial resolution of 0.083° across 30 years as satellite‐derived reconstructions of sea currents (layers originally from the ‘GLOBAL_REANALYSIS_PHY_001_030_104’ dataset, accessed on 03‐04‐2024; CMEMS 2024). For each pixel, we calculated the cumulative speed towards each of the eight neighbouring pixels and divided this by the total speed to obtain a probability of transition in each direction (the conductance). We then calculated the dispersal costs as the inverse of the square conductance to obtain transition matrices using the *gdistance* R package (v1.6.4, van Etten 2017). Finally, we calculated the shortest sea distance (least‐cost path) between pairs of sites in both directions from the transition matrix to obtain an asymmetrical square matrix of shortest sea distance. IBR models were built for the mean monthly and annual sea currents, resulting in 13 IBR models. The explanatory power of IBD and IBR models were evaluated using leave‐one‐reef‐out jackknife on linear regressions of linearised *F*_ST_ against distance (Euclidean or sea distance; 14 models total). Linear model performance was evaluated using coefficients of determination (*R* ^2^), with differences between IBD and each IBR model assessed using paired Wilcoxon signed‐rank tests across jackknife iterations, correcting for multiple comparisons using false discovery rate. The significance of the best performing model for each species was tested with a Mantel test through the *vegan* R package (v.2.6–8; Oksanen et al. 2024).

### 2.8 Genotype–Environment Associations (GEA)

We performed GEAs using multivariate redundancy analyses (RDA) at the site‐level following methods of Capblancq and Forester (2021). For each sampling site, we converted individual genotypes of the filtered dataset to allele frequencies per locus by averaging observed genotypes across individuals, ignoring missing data, with data from 4 to 27 individuals per site for *A. muricata* and 2–12 individuals per site for *P. damicornis* (Table S1). We imputed allele frequencies for loci missing information at the site‐level using the median allele frequency from the other sites in the same region (i.e., Seychelles, Mauritius or Rodrigues), where loci missingness at any one site was < 0.2% ( *A. muricata* : site S04 had 0.06% loci missing allele frequency values, S05 0.01%, S07 0.17%; *P. damicornis* : S01 0.10%, S04 0.01%, S09 0.02%, S11 0.02%, S12 0.20%). The final set of environmental predictors included in the explanatory matrix were selected from the 10 uncorrelated variables (Table S2) using a bidirectional stepwise model selection based on permutation tests (10,000 permutations) implemented with the *ordistep* function in *vegan*. Variables were sequentially added or removed according to their contribution to model fit, retaining only those that significantly improved the explained neutral genetic variation (*p* < 0.05). The stepwise procedure retained sSST (*F* = 10.86, *p* < 0.001) and mDHW (*F* = 5.51, *p* = 0.0011) for *A. muricata* , and sSST (*F* = 6.07, *p* < 0.001), mDHW (*F* = 2.76, *p* = 0.003), and BackReef (*F* = 1.91, *p* = 0.006) for *P. damicornis* . The percentage of neutral genetic variance explained per selected variable was assessed using the *varpart* function in *vegan*.

To detect putative genomic regions under selection, we performed a multivariate GEA using a partial RDA (pRDA) on the entire genotype matrix, with the *ordistep* selected variables as predictors. Reef‐level population structure was used to condition the RDA, limiting false positives while potentially reducing power to detect true outlier loci along neutral gradients (Excoffier et al. 2009; Lotterhos 2023). In this way, we expect the outlier loci from the pRDA to represent genetic variants repeatedly associated with environmental conditions across the populations while removing variants resulting from structuring. Population structure was accounted for using the first *K* PC axes that explained more than the mean explained variance in a PCA on the centred and scaled neutral allele frequencies, using the *rda* function in *vegan*.

Outlier loci were identified from RDA loadings (Capblancq et al. 2018). After retaining the first two constrained RDA axes, we evaluated the significance of each SNP based on the extremeness of its Mahalanobis distance value compared to the distribution of the other SNPs in the RDA space. The Mahalanobis distances were computed using the *dist_ogk* function of the *bigutilsr* R package (v.0.3.4; Privé 2021), corrected for genomic inflation factor (François et al. 2016) and distributed along a chi‐squared distribution with *K* degrees of freedom to assign a *p*‐value to each SNP (Luu et al. 2017). We applied an FDR threshold of *q* < 0.05 to identify outlier loci.

### 2.9 Gene Ontology (GO) Enrichment Analysis

Gene Ontology (GO) enrichment analyses were used to assess the putative molecular function(s) of significant outlier SNPs (i.e., *q* < 0.05) from the pRDA, following Selmoni et al. (2021). To facilitate comparability of annotations between species, we re‐annotated genes in both reference genomes. For this, we retrieved transcript sequences of each gene, then ran a similarity search (blastx) against a manually curated database of protein sequences and annotations (Uniprot/swissprot, metazoan entries as accessed on November 2022; Boeckmann et al. 2003). Protein annotations were assigned to coral genes when the E‐score of the similarity search was < 10^−6^. We then identified genes located within ±10 kbp of significant SNPs of the GEA analyses (hereafter ‘significant genes’; Selmoni et al. 2021). We evaluated gene‐set enrichments of significant genes using the *SetRank* R package (version 1.0; Simillion et al. 2017), where genes are ranked based on ascending *q*‐values from the GEA, before identifying overrepresented GO terms associated with significant genes. GO terms were deemed significant when they had *SetRank p* < 0.05 and adjusted *p* < 0.05 (i.e., corrected for multiple testing and overlap between gene‐set categories).

### 2.10 Estimating the Adaptive Seascape

We estimated the adaptive seascape across the WIO to identify reefs potentially harbouring adaptive genetic variation linked to thermal gradients. For each species, we performed an RDA to quantify genotype–environment interactions using site‐level allele frequencies as the response matrix. We used variables selected in Section 2.8 as the prediction matrix: sSST and mDHW for *A. muricata* ; sSST, mDHW and BackReef for *P. damicornis* . We included all loci in the RDA to capture the full genetic variation and polygenic nature of thermal adaptation, following recommendations of Lotterhos (2023). The resulting RDA characterises how multivariate allele frequencies covary with environmental gradients at the study sites. We then projected environmental conditions from all mapped WIO reefs into the RDA ordination space to derive a genetic‐based index of adaptation (Adaptive Index) for each environmental pixel of the seascape (Steane et al. 2014). This Adaptive Index reflects how genetic variation is structured relative to thermal gradients, allowing us to map the spatial distribution of reefs where allelic compositions are most consistent with thermal adaptive potential across the WIO seascape (Capblancq et al. 2020; Capblancq and Forester 2021). We overlaid these maps with the mean ocean current velocities in January to aid with interpretations. We quantified up‐ and down‐current reef connectivity in January for each reef pixel in the WIO using an ‘Inbound Connectivity Index’ (ICI) and ‘Outbound Connectivity Index’ (OCI), respectively (described in detail in Selmoni, Vajana, et al. 2020). Briefly, these connectivity indices represent the sum of reef area either up‐ or down‐current, respectively, from a target reef within a set sea distance threshold. We chose the sea distance threshold that corresponds to the maximum regional *F*_ST_ values (Figure S3), with sea distance values obtained from the linear relationship calculated between log of sea distances in January with linearised *F*_ST_ (from Section 2.7). In this way, ICI and OCI provide a spatial summary of how reefs across the region are susceptible to receive or send propagules, based on sea currents and reef area.

## 3 Results

### 3.1 Site Characteristics and Genomic Filtering

We selected 15 reefs for coral sampling: six sites in the Seychelles (three each at Mahé and Praslin), six in Mauritius and three in Rodrigues (Figure 1a). These reefs represent contrasting environmental conditions (Figure 1b), characterised by 10 uncorrelated environmental variables (Table S2; Figure S2). The Seychelles (represented in red) were the northern‐most sampled reefs, characterised by back reefs located near deep water with reduced thermal stress (i.e., low sSST and low mDHW), and generally further from human activity (i.e., cropland, human population and boat activity). The south‐western reefs of Mauritius (represented in blue) were characterised by reef flats near land and human activity (i.e., cropland and boats) and experienced more variable thermal conditions. However, the northern‐most Mauritian reef at Ile Plate (S12 in dark blue) was characterised by deeper waters and more stable temperatures, away from human activity. Finally, the south‐eastern reefs of Rodrigues (represented in green) were predominantly isolated reef flats, located near deeper waters that experience hotter (higher mDHW) and more variable SST conditions (sSST).

Between 8 and 29 individuals per species were sampled for genotyping at each site (Figure 1a; Table S1). The DArT‐seq analytical pipeline resulted in 73,253 bi‐allelic SNPs genotyped for 345 *A. muricata* individuals and 65,708 SNPs for 403 *P. damicornis* individuals (counts of individuals and loci retained at each filtering step are summarised in Table S4). We removed 88 *A. muricata* and 43 *P. damicornis* individuals identified as putatively cryptic based on PCoA biplots to group genetically distinct individuals sampled at the same sites (Grupstra et al. 2024; Riginos et al. 2024; Figure S3). These distinct groups had pairwise *F*_ST_ > 0.3 when compared with the retained genetic groups (Figure S3).

We removed 22 *A. muricata* and 252 *P. damicornis* individuals identified as clones (i.e., individuals sharing > 95% and > 90% of their genomes, respectively). For *A. muricata* , clones were most frequently sampled in the southern reefs (Mauritius and Rodrigues), whereas clonal colonies of *P. damicornis* were present across all reefs (Figure S4). Most clonal group clusters were found within regions ( *A. muricata* : 11 groups; *P. damicornis* : 49 groups), with some groups spanning across regions (*A. muricata*: 3 groups; *P. damicornis* : 13 groups)—likely due to errors in sampling labelling or DNA contamination. Following the final filtering step that removed loci and individuals missing > 80% of information and excluded rare variants (MAF < 0.05), our final genotype matrix comprised of 211 *A. muricata* genotypes with 7663 SNPs (approximately 1 SNP per 55 kbp for an estimated genome size of 420.7 Mbp; Shinzato et al. 2021) and 97 *P. damicornis* genotypes with 13,190 SNPs (approximately 1 SNP per 25 kbp for an estimated genome size of 349 Mbp; Cunning et al. 2018). This resulted in 4–27 *A. muricata* individuals retained per site (note that no samples were left at S06 Curieuse Reef, Seychelles), and 2–12 *P. damicornis* individuals retained per site (note that no samples were left at S07 Coco Reef, Seychelles; Table S1).

### 3.2 Population Structure Reflects Geographic Distribution

Outlier loci were identified and removed using a genome scan via the *pcadapt* R package to produce a putatively neutral genotype matrix (hereafter termed ‘neutral genotype matrix’) for assessing WIO population structure. We retained *K* = 2 PCs for the genome scan, identifying 124 and 103 outlier SNPs (*q* < 0.05) for *A. muricata* and *P. damicornis* , respectively. This resulted in respective neutral genotype matrices comprising 7539 and 13,087 SNPs each (Table S4).

**FIGURE 2:**
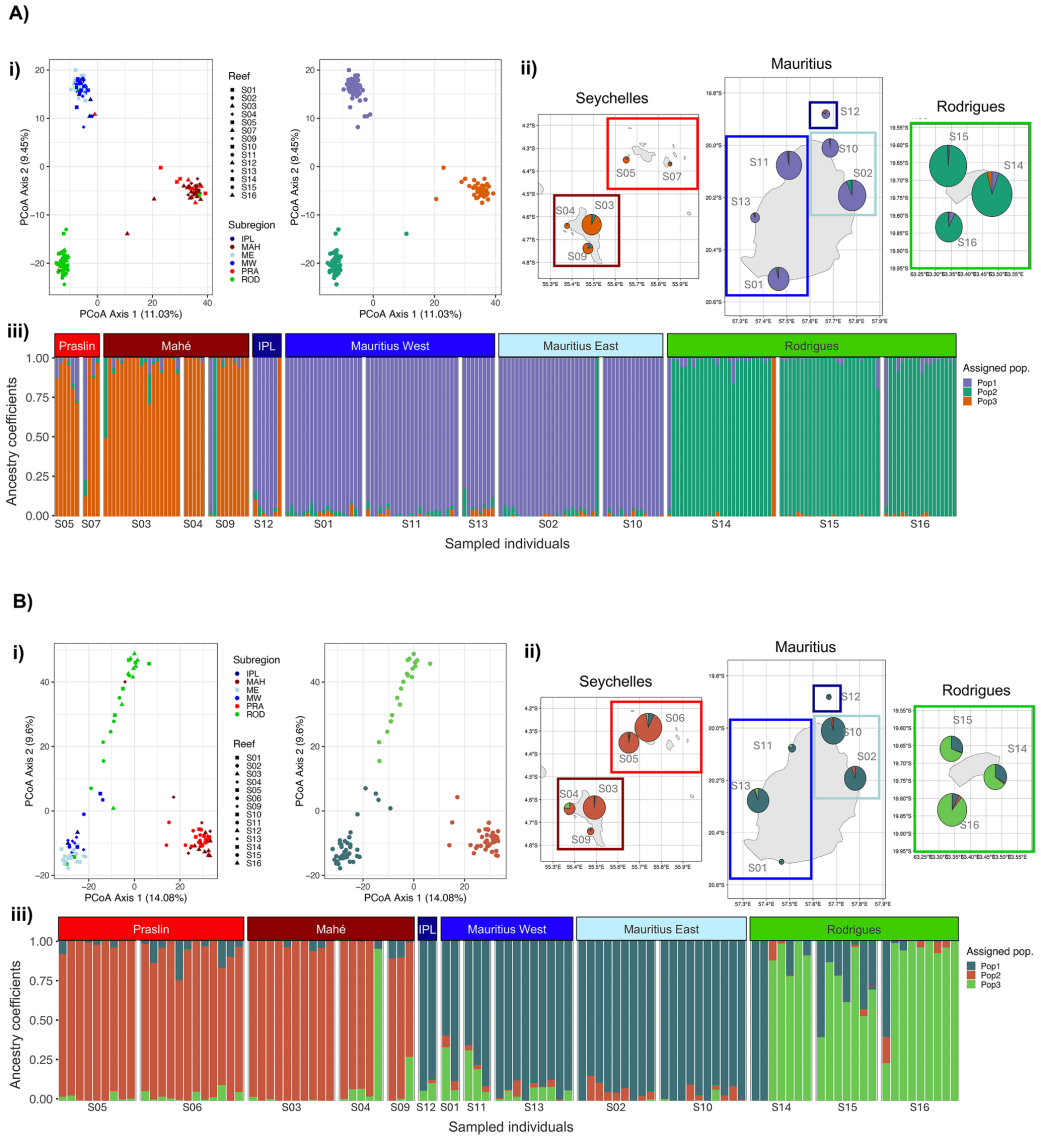
Population structure analyses of (A) *A. muricata* and (B) *P. damicornis* , assessed by a principal coordinate analysis (PCoA) and sparse nonnegative matrix factorization (sNMF) on a neutral filtered genomic dataset, where *K* = 3 genetically distinct groups were identified for each species using both methods. (i) Individual scores on PCoA axes 1 and 2 grouped by geographic location (shapes indicate reef and colours indicate subregion; left) and hierarchical cluster grouping of PCoA results (coloured by genetically‐assigned grouping; right). (ii) Maps indicating the proportion of individuals at each sampled reef allocated to *K* ancestral populations using sNMF admixture models, where pie chart size indicates the relative number of genotyped individuals at each reef with coloured boxes indicating sub‐regions. (iii) Proportion of genotyped individuals (columns) allocated to *K* ancestral populations using sNMF admixture models, with individuals grouped by sub‐region. Sub‐region abbreviations: IPL = Ile Plate (Mauritius), MAH = Mahé (Seychelles), ME = East Mauritius, MW = West Mauritius, PRA = Praslin (Seychelles), ROD = Rodrigues.

We found strong regional population structuring for both species, where PCoA and sNMF models indicate the presence of three genetic clusters strongly associated with the three regions of Rodrigues, Mauritius and Seychelles (Figure 2). For *A. muricata*, the first two PCoA axes (capturing 20.5% of neutral genetic variation) assigned individuals into separating reefs of north–south for Axis 1 and Mauritius‐Rodrigues for Axis 2 (Figure 2a[i]). sNMF models confirmed that individuals were more genetically similar within regions and more different between regions, with some individuals exhibiting evidence of admixture across regions (Figure 2a[ii,iii]). These patterns are statistically supported by AMOVAs, where genomic variance is significantly higher between regions than expected by chance (*p* < 0.01; Figure S5a). In contrast, variance within subregions was not significant (*p* = 0.24). Low pairwise *F*_ST_ values ≤ 0.02 further support genetic similarity between reefs in a region (Table S5). In contrast, *F*_ST_ values between the Seychelles and the two southern regions exceeded 0.20 in most pairwise comparisons, signalling increased genetic divergence between more distant regions (Table S5).

Similar genetic patterns were identified for *P. damicornis* . Three genetic clusters associated with the geographic regions were identified using PCoA (first two axes capturing 24% of neutral genetic variance) and sNMF (Figure 2b). The first PCoA axis separates the Seychelles from the southern reefs (Figure 2b[i]), while the second PCoA axis and sNMF plots indicate some admixture between individuals from Rodrigues and Mauritius. Within regions, individuals appeared more genetically similar (pairwise *F*_ST_ < 0.10), while *F*_ST_ ≥ 0.20 between the Seychelles and the southern reefs suggests large genetic differences (Table S5). AMOVA statistics highlight significant genomic variance between regions (*p* = 0.01; Figure S5b).

### 3.3 Isolation‐By‐Resistance Best Explains Patterns in Population Structure

**FIGURE 3:**
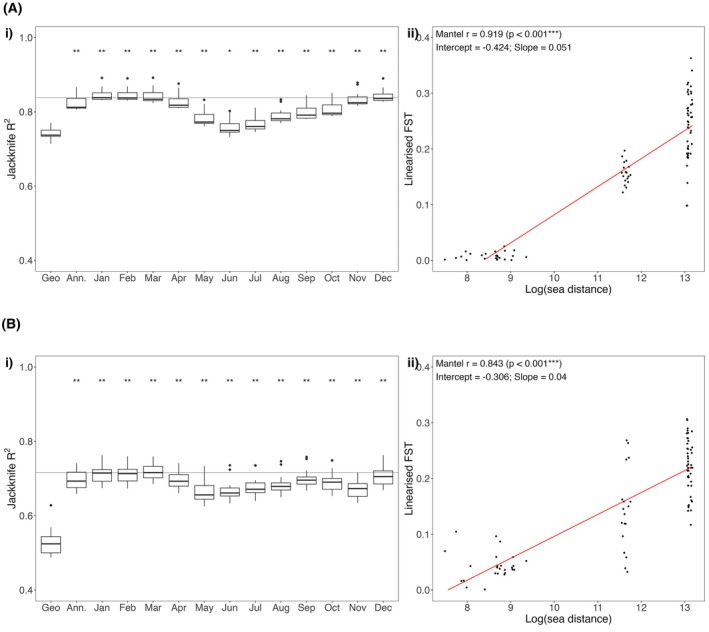
Isolation‐by‐distance (IBD; Euclidean distances) and isolation‐by‐resistance (IBR; sea currents) as predictors of genomic distance (measured as linearised *F*_ST_) between sample sites for (A) *A. muricata* and (B) *P. damicornis* . (i) Predictive performance of IBD and IBR assessed by regressing linearised *F*_ST_ against log_10_ of the geographic distance for IBD (represented by ‘Geo’) or log_10_ of sea distance (IBR) calculated from mean annual (‘Ann.’) or monthly (‘Jan’ to ‘Dec’) ocean current data. Boxplots of model performance (*R* ^2^) show the spread of the interquartile range (IQR) obtained from leave‐one‐reef‐out jackknifing, with whiskers extending to 1.5 × IQR and outliers as points. Asterisks above boxplots denote the significance of paired Wilcoxon signed‐rank tests comparing each IBR model with IBD (where *p < 0.05, **p < 0.01 after correcting for false discovery rate). The largest of the median *R* ^2^ for each species is indicated by a grey horizontal line across the boxplots. (ii) IBR relationship between linearised genetic distance with one of the optimal genetic predictors, sea currents in January, with IBR significance from Mantel tests provided in the insert.

Patterns of IBR were significantly stronger than IBD for both species. For *A. muricata* , monthly sea currents explained a median of 81% of the variance in genetic distance (measured as linear *F*_ST_; IBR median *R* ^2^ = 0.75–0.84 across all monthly current models), assessed using a leave‐one‐reef‐out jackknife analysis (Figure 3a[i]). Sea currents during the spawning period (December to February) were the best predictors of *F*_ST_ (median *R* ^2^ = 0.84, IQR 0.83–0.85) and were each significantly better predictors than IBD (pairwise Wilcoxon tests, *p*‐adjust = 0.0013), where IBD explained on median 74% of the genetic variance (IQR 0.73–0.75). Similar, albeit weaker, patterns of IBR and IBD were observed for *P. damicornis* (Figure 3b[i]). IBR calculated from monthly sea currents explained a median of 69% of the variance in genetic distance (IBR median *R* ^2^ = 0.66–0.72 across all monthly current models). There was a slight increase in explanatory power of sea currents from January to March (median *R* ^2^ = 0.72, IQR = 0.69–0.73), which were all significantly better predictors than IBD that had an *R* ^2^ = 0.52 (IQR 0.50–0.54; pairwise Wilcoxon tests, *p*‐adjust = 0.0011).

We further investigated IBR using mean sea currents during the coral spawning month of January, as this was one of the top predictors of genetic distance for both species ( *A. muricata* : median *R* ^2^ = 0.84, IQR: 0.83–0.85; *P. damicornis* : median *R* ^2^ = 0.72, IQR: 0.69–0.72). Mantel tests of significance for IBR using January currents were significant for both species ( *A. muricata* : *r* = 0.919, *p* < 0.001; *P. damicornis* : *r* = 0.843, *p* < 0.001). The strength of the IBR relationship reflected the observed patterns of population structure and known reproductive modes, where the broadcast spawning coral of *A. muricata* had a slightly steeper IBR slope by approximately 1.3 times than *P. damicornis* with mixed reproductive modes (*m* = 0.051 vs. 0.040; Figure 3a[ii],b[ii]).

### 3.4 Genotype–Environment Associations Identified Putative Loci Under Selection

Among the ten variables used to characterise the reef environment, factors relating to thermal stress emerged as the best predictors of reef‐level neutral genetic variation in both *A. muricata* and *P. damicornis* . For *A. muricata*, sSST (*F* = 10.85, *p* = 0.0003) and mDHW (*F* = 5.51, *p* = 0.0011) were selected as top variables, capturing 43.1% and 29.0% of explained neutral genetic variation, respectively. For *P. damicornis* , sSST (*F* = 6.07, *p* < 0.001), mDHW (*F* = 2.76, *p* = 0.003), and BackReef (*F* = 1.91, *p* = 0.006) were selected, respectively capturing 28.1%, 14.0% and 6.4% of neutral genetic variation.

**FIGURE 4:**
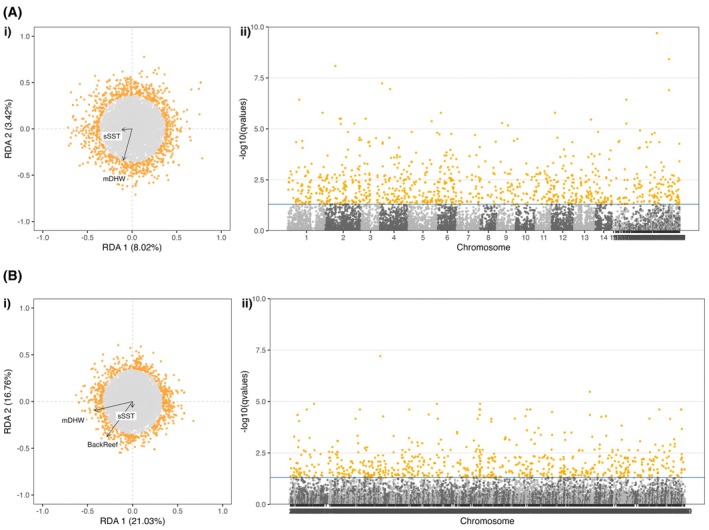
Genotype–environment association (GEA) results for (A) *A. muricata* and (B) *P. damicornis* , calculated from multivariate pRDA (partial redundancy analyses) of allele frequencies at the sampled reefs associated with selected environmental variables, while controlling for neutral population structure. (i) RDA biplots projecting loci (points) and environmental variables (arrows; BackReef = proportion of back reef around sample site; mDHW = mean degree heating weeks; sSST = standard deviation of sea surface temperature). Locus scores are rescaled by a factor of 15 to improve visibility. (ii) Manhattan plots highlight the distribution of loci significance (as −log_10_(*q‐*values)) across the genomes (chromosomes indicated by alternating light and dark grey dots). Loci identified as outliers (*q* < 0.05) are coloured in orange in both panels.

We performed multivariate GEAs using a pRDA that conditioned on the strong patterns of neutral population structure, with the aim of reducing false positive rates when detecting outlier loci (Forester et al. 2018). Population‐level structure was accounted for using 3 PCs for *A. muricata* (explaining 69.0% variance) and 4 PCs for *P. damicornis* (explaining 64.7% variance). For *A. muricata* , the first and second pRDA axes respectively explained 8.0% and 3.4% of genetic variation (Figure 4a[i]). Population structure (PC1) was negatively correlated with sSST (*r* = −0.98), thus reducing its association with the RDA axes. RDA1 was negatively associated with both mDHW and sSST, while RDA2 was negatively associated with mDHW. A total of 901 outlier loci were identified (*q* < 0.05) throughout the genome (Figure 4a[ii]). SetRank analyses on 1564 significant genes (i.e., those located within ±10 kbp of significant SNPs) revealed 12 enriched GO terms (molecular functions) associated with 92 significant genes (Tables 1a and S6).

For *P. damicornis* , the first and second pRDA axes respectively explained 21.0% and 16.8% of genetic variation (Figure 4b[i]). Population structure (PC1) was again negatively correlated with sSST (*r* = −0.97), reducing its association with the RDA axes. RDA1 was negatively associated with mDHW and BackReef, while RDA2 was negatively associated with BackReef. The analysis identified 690 outlier loci throughout the genome (Figure 4b[ii]). SetRank enrichment analyses on 1319 significant genes revealed 20 enriched molecular functions associated with 85 significant genes (Tables 1b and S6).

**TABLE 1:** Summary of SetRank Gene Ontology (GO) term enrichment analysis performed on candidate loci identified from multivariate GEAs for (A) *A. muricata* and (B) *P. damicornis* . The table lists the significantly enriched GO terms of molecular function (*p* < 0.05) with their functional descriptions, adjusted *p‐*values, gene set size, and the number of associated significant genes. Detailed GO term results, including the names of associated genes, setRank scores and setRank *p*‐values, are provided in Table S6.

(A)				
GO term	Description	Adjusted *p*‐value	Gene set size	Associated significant genes
GO:0022852	Glycine‐gated chloride ion channel activity	< 0.001	4	2
GO:0005516	Calmodulin binding	0.004	73	14
GO:0004687	Myosin light chain kinase activity	0.004	2	2
GO:0019901	Protein kinase binding	0.004	113	26
GO:0004674	Protein serine/threonine kinase activity	0.004	96	26
GO:0051015	Actin filament binding	0.004	64	14
GO:0004563	Beta‐N‐acetylhexosaminidase activity	0.006	6	4
GO:0030247	Polysaccharide binding	0.006	5	4
GO:0015280	Ligand‐gated sodium channel activity	0.017	18	9
GO:0045174	Glutathione dehydrogenase (ascorbate) activity	0.018	2	2
GO:0051087	Chaperone binding	0.022	28	12
GO:0070628	Proteasome binding	0.022	11	7

(B)				
GO term	Description	Adjusted *p*‐value	Gene set size	Associated significant genes
GO:0005102	Signalling receptor binding	0.007	116	13
GO:0019210	Kinase inhibitor activity	0.007	3	2
GO:0015101	Organic cation transmembrane transporter activity	0.007	3	2
GO:0019534	Toxin transmembrane transporter activity	0.007	6	4
GO:0008289	Lipid binding	0.012	79	16
GO:0005178	Integrin binding	0.013	76	16
GO:0004831	Tyrosine‐tRNA ligase activity	0.013	1	1
GO:0070579	Methylcytosine dioxygenase activity	0.013	2	2
GO:0000049	tRNA binding	0.013	27	6
GO:0002196	Ser‐tRNA(Ala) hydrolase activity	0.013	1	1
GO:0106105	Ala‐tRNA(Thr) hydrolase activity	0.013	1	1
GO:0016597	Amino acid binding	0.013	15	4
GO:1990984	tRNA demethylase activity	0.013	1	1
GO:0034186	Apolipoprotein A‐I binding	0.041	1	1
GO:0042626	ATPase‐coupled transmembrane transporter activity	0.041	43	6
GO:0090556	Phosphatidylserine floppase activity	0.041	3	3
GO:0140346	Phosphatidylserine flippase activity	0.041	2	2
GO:0016887	ATP hydrolysis activity	0.041	152	28
GO:0120020	Cholesterol transfer activity	0.041	7	2
GO:0140481	ABC‐type iron–sulfur cluster transporter activity	0.041	1	1

### 3.5 Adaptive Seascape Patterns Across the Western Indian Ocean

We estimated the adaptive seascape across the WIO to obtain species‐specific maps highlighting reefs that may harbour coral individuals adapted to thermally stressful environments. These maps were produced using multivariate RDAs (without structure correction), where the thermal history and allele frequency interactions were combined to produce an Adaptive Index at novel sites. Interpretations of connectivity between reefs were quantified using inbound (ICI) and outbound (OCI) connectivity indices, which represent the area (km^2^) of reefs either up‐ or down‐current, respectively, from a target reef within a set distance threshold corresponding to the maximum regional *F*_ST_ values (derived from IBR linear models; Figure 3[ii]). The maximum regional *F*_ST_ = 0.014 for *A. muricata*, corresponding to a distance threshold of 5815 km, while regional *F*_ST_ = 0.067 for *P. damicornis*, corresponding to 11,189 km.

**FIGURE 5:**
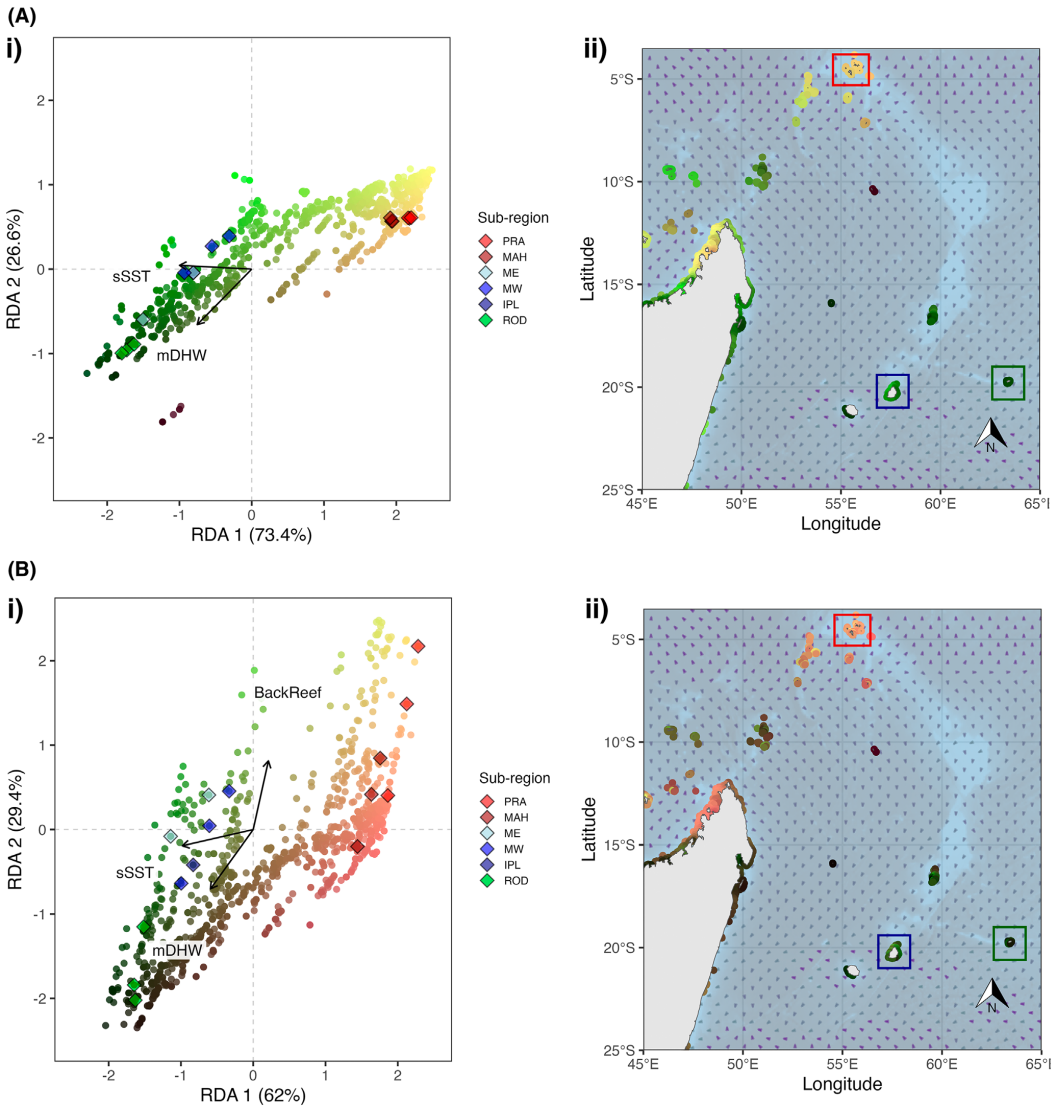
Adaptive seascape across the Western Indian Ocean (WIO) for (A) *A. muricata* and (B) *P. damicornis* , calculated from multivariate RDA (redundancy analyses) of allele frequencies at the sampled reefs associated with selected environmental variables. (i) RDA projections for each WIO reef (coloured dots) across RDA axes 1 and 2 according to environmental conditions (arrows; BackReef = proportion of back reef around sample site; mDHW = mean degree heating weeks; sSST = standard deviation of sea surface temperature). Sample sites used to build RDA are indicated as diamonds coloured by sub‐region. Sub‐region abbreviations: IPL = Ile Plate (Mauritius), MAH = Mahé (Seychelles), ME = East Mauritius, MW = West Mauritius, PRA = Praslin (Seychelles), ROD = Rodrigues. (ii) Spatial projection of RDA‐predicted adaptive genotypes across the WIO, with reef colours corresponding to those in panel (i). The direction of ocean currents in January are shown by the direction of the arrow heads, while darker arrows indicate the relative strength of the currents. Sampled reef regions are outlined with boxes coloured by region, as per Figure 1: red = Seychelles, blue = Mauritius, green = Rodrigues.

Genomic patterns across the study sites were strongly associated with differences in thermal conditions for *A. muricata* , and mapping reef environmental conditions from the whole WIO into this RDA space identified two groups of reefs with contrasting adaptive potentials (Figure 5a[i]). First are reefs with negative values along RDA axes 1 and 2 (dark green points in Figure 5a). These are associated with high thermal variability (sSST) and accumulating heat stress (mDHW), and identify reefs more likely to harbour thermally adaptive alleles. This bottom‐right corner of the ordination space is driven by sampled sites in the southern reefs of Mauritius and Rodrigues, and predicts that reefs at Réunion and east Madagascar might also have similar allelic compositions (Figure 5a[ii]). January ocean currents indicate that gene flow might occur in a westerly direction (Figure 5a[ii]), with sampled sites at Mauritius and Rodrigues connected to approximately 24–150 km^2^ of reefs down current (Figure S6a; Table S7). The second group are those positively associated with RDA axes 1 and 2 (yellow or light orange points in Figure 5a), where they experience less stressful thermal conditions and are less likely to harbour thermal‐adaptive allele compositions. These reefs include the Seychelles and northern Madagascar reefs, which do not appear to be down current for receiving propagules from putative heat‐adapted reefs (Figure 5a[ii]), where sampled sites at the Seychelles are estimated to be connected to only 2–45 km^2^ of neighbouring reefs (Figure S6a; Table S7).

Genomic variation of *P. damicornis* was spread across both RDA axes, revealing three groups of reefs when novel present‐day WIO environmental conditions were mapped into the ordination space (Figure 5b[i]). The first group was driven by study sites at Mauritius and Rodrigues, with negative values on RDA axes 1 and 2 associated with high thermal variability and stress, but low proportion of back reef (dark green points in Figure 5b). These reefs putatively harbour thermal‐adaptive alleles driven by region‐scale thermal anomalies and are estimated to be distributed across the southern WIO around Rodrigues, Mauritius, Réunion and east Madagascar (Figure 5b[ii]). We estimate that these sampled sites at Mauritius and Rodrigues are connected to approximately 71–270 km^2^ of reefs down‐current (Figure S6b; Table S7). The second group of reefs had high values on RDA axes 1 and 2 (yellow points in Figure 5b), characterised by a high proportion of back reef. These reefs appear to be sporadically distributed around the Seychelles (notably Praslin) and Mayotte to the west of Madagascar (Figure 5b[ii]), where corals likely experience high daily temperature fluctuations that could provide thermal adaptation to reef‐scale conditions. Intermingled on the map are the third group of reefs with high RDA axis 1 values and values close to zero on RDA axis 2 (pink points in Figure 5b), indicating reefs of low thermal stress at either the regional or reef levels, and as such are less likely to be thermally adapted to future conditions. Ocean currents in January indicate that there may be potential for genetic exchange amongst these last two groups (yellow and pink points), with sampled *P. damicornis* at BackReef sites estimated to contribute propagules to 27 km^2^ of down‐current reefs, while reefs expected to host non‐thermal adapted corals could receive propagules from 4 to 20 km^2^ of other reefs (Figure S6b; Table S7).

## 4 Discussion

### 4.1 Life‐History Traits and Asymmetric Ocean Currents Drive WIO Population Structure

Coupling genomic information with environmental data revealed the importance of coral life‐history strategies in driving spatial patterns of population structure across the WIO, likely affected by asymmetrical ocean currents. Population structuring of both species corroborates connectivity simulations and mapped genetic structuring of various other coral species across the WIO (e.g., Gélin et al. 2018; Kusumoto et al. 2020; Oury et al. 2024; Vogt‐Vincent et al. 2024). Furthermore, we confirm the hypothesis that large breaks in reef connectivity (> 100 km of open sea) are associated with stronger population structuring across the WIO for both species, where we find evidence of genetic exchange across further distances for *P. damicornis* than for *A. muricata* .

*A. muricata* showed strong patterns of local genetic mixing with large genetic differences between regions of the WIO, mirroring expectations for a synchronous broadcast spawning coral species (Miller and Ayre 2008). Indeed, high gene flow between reefs of a region within a relatively short distance is observed for numerous Acroporidae around the world (e.g., Adam et al. 2022; Fuller et al. 2020; Lukoschek et al. 2016; Nakajima et al. 2010; Selmoni et al. 2021). Genetic mixing is promoted by synchronous release of gametes (Wijayanti et al. 2019), while a free‐swimming larval stage of approximately 2 weeks allows propagules to disperse to neighbouring reefs (Tabitha et al. 2021), though not beyond. Here, we found that corals of the southern Mascarene Island reefs (Mauritius and Rodrigues) are more genetically similar based on a PCoA, while northern corals in the Seychelles are more genetically isolated.

Similar population structuring was found for *P. damicornis* . PCoA biplots indicate distinct genetic grouping associated with the three regions. However, we found more genetic similarity amongst individuals in the southern reefs for *P. damicornis* individuals than we did for *A. muricata* , reflecting patterns of *P. damicornis* Type β dispersal across the southern WIO (Gélin et al. 2018). Genetic exchange between these reefs > 600 km apart could be explained by the mixed reproductive mode of *P. damicornis* . High rates of asexual reproduction via colony fragmentation (evident from the large number of clones identified among our samples) or brooding larvae with up to 14 weeks viability (Gélin et al. 2017, 2018; Richmond 1987) means that propagules could survive passively travelling long distances, either along ocean currents (Phillips et al. 2021), by rafting (Nikula et al. 2013), or in boat ballast water (Gollasch 2008).

Reproductive mode plays an important role in shaping coral demographic patterns, yet population structure is rarely a simple function of reproductive mode or larval type (Miller and Ayre 2008; Prata et al. 2024; Severance and Karl 2006). The present study is limited in its conclusions regarding demographic patterns between reefs, where the use of *F*_ST_ to gain insights into population patterns cannot be extrapolated to infer gene flow or migration rates (reviewed in Meirmans and Hedrick 2011; Whitlock and Mccauley 1999). Combining population structure patterns with past research into ocean currents and coral demography across the WIO suggests that genetic exchange for our study species in the southern WIO is likely driven by the strong South Equatorial Current that cuts westerly across the WIO at a latitude of 10°S (Phillips et al. 2021), moving from Rodrigues towards the other Mascarene Island reefs and potentially beyond to Madagascar and the African east coast (Vogt‐Vincent et al. 2024). This same oceanographic barrier likely prevents genetic exchange between southern and equatorial reefs, with important consequences for the spread of adapted genotypes across the WIO, as well as subsequent design and implementation of regional management strategies (Phillips et al. 2021; Vogt‐Vincent et al. 2024). More widespread genetic sampling of corals and explicit demographic modelling is required to draw precise conclusions of population structure and patterns of gene flow across the WIO region.

### 4.2 Sea Currents as a Proxy for Genetic Connectivity and Dispersal

Isolation‐by‐resistance (IBR), measured using sea currents to derive asymmetric distance between reefs, was a better predictor of population connectivity than Euclidean‐derived isolation‐by‐distance (IBD) for both coral species. This finding supports prior research into coral dispersal along asymmetric currents, typically during the pelagic larvae stage (Carlon 1999) or via rafting (Jokiel 1984). We show that *A. muricata* genetic distance was most strongly predicted by currents in November to February, coinciding with this species' known mass spawning events in the Austral summer (Wijayanti et al. 2019). By contrast, *P. damicornis* showed weaker patterns of IBR by month, potentially driven by its high rates of clonal fragmentation and asexually brooded larvae that can travel longer distances by currents throughout the year. The slight improvement in predictive ability of currents in the Austral summer for *P. damicornis* could be due to stronger ocean currents during that season (Vogt‐Vincent et al. 2024).

The correlation (*r* ^2^) and regression (*m*) coefficients of the final linear IBR models can provide information on the importance of sea currents to disperse propagules across reef networks. For example, January currents produced IBR with *r* ^2^ = 0.84 for *A. muricata* and *r* ^2^ = 0.72 for *P. damicornis* . IBR slope (*m*) steepness may indicate the strength of ocean currents acting as a barrier to gene flow. Here, average January currents resulted in 1.3 times steeper regression coefficient for *A. muricata* than *P. damicornis* (*m* = 0.051 vs. 0.040), which may be explained by the length of the larval stage and consequent dispersal potentials (3 vs. 14 weeks; Richmond 1987; Tabitha et al. 2021). We note that slope steepness of IBR and IBD could be reflecting effective local population sizes (Slatkin 1987), which would need to be investigated in future analyses. Overall, these metrics could generate hypotheses of dispersal strength and life history strategies to facilitate the development of management strategies for non‐model coral species.

### 4.3 Historical Thermal Stress as a Putative Driver of Local Adaptation

Environmental variables capturing historical thermal variation and accumulated heat stress were the strongest predictors of genetic diversity for both coral species across the WIO. Multivariate genotype–environment associations (GEAs) detected 901 and 690 outlier loci for *A. muricata* and *P. damicornis* , respectively, as significantly associated with sea surface temperature variation (sSST; Skirving et al. 2020) and cumulative thermal stress (mDHW; Skirving et al. 2020). For *P. damicornis* , an additional association was detected with the proportion of back reef (BackReef), which, while weakly negatively correlated with proportion of reef flat, sSST and mDHW at coarse resolutions, captures fine‐scale areas of stagnated water around reefs (Dahlgren and Marr 2004) with highly varying daily thermal fluctuations (Rogers et al. 2016). These genotype–environment interactions appear to be driven by differences between southern and northern reefs for both species, which are strongly characterised by thermal environments (Figure 1). For instance, corals in the south‐eastern reefs at Rodrigues are characterised by reef flats that seem to experience more varying temperatures and accumulated heat stress. In contrast, the Seychelles are generally associated with cooler annual temperatures and lower accumulated thermal stress, though they have more back reefs that could result in larger daily temperature fluctuations (Dahlgren and Marr 2004).

Extreme and variable marine thermal conditions can trigger coral bleaching when SST fluctuations exceed seasonal averages (Hughes, Kerry, et al. 2017; Liu et al. 2003), as seen across the Indian Ocean (Shlesinger and van Woesik 2023). With recurrent mass‐bleaching events exerting strong selective pressures on corals (Hughes, Kerry, et al. 2017; Skirving et al. 2020), affected populations are expected to have molecular signatures of thermal adaptation and tolerance (Lachs et al. 2023). Here, we see large numbers of candidate loci associated with regional variations of annual SST and mean DHW at 5‐km resolutions, where these variables capture both the duration and intensity of broad scale thermal anomalies often associated with bleaching events (Humanes et al. 2022; Liu et al. 2003; Skirving et al. 2020; van Hooidonk et al. 2016). Simultaneously, microhabitat variation across reefs at spatial scales < 100 m is expected to influence patterns of adaptations (e.g., Bay and Palumbi 2014; Bozec et al. 2025). Here, we detected the proportion of back reef as an important predictor of *P. damicornis* adaptive genetic variation, serving as a high‐resolution proxy for exposure to heat stress. This finding supports previous studies identifying divergent SNPs under thermal selection across back reef habitats within single reef systems (Bay and Palumbi 2014; Thomas et al. 2018), and highlights the importance of considering finer scale processes.

### 4.4 Candidate Molecular Targets of Thermal Stress

Genotype–environment associations and subsequent GO term enrichment analyses for significant genes (i.e., those located within 10 kbp of significant SNPs) revealed molecular functions in both species previously associated with coral thermal responses. We detected 12 molecular functions enriched for *A. muricata* pertaining to signal transduction and calcium‐mediated regulation, cytoskeletal organisation, protein folding and degradation, redox homeostasis, ion transport, and carbohydrate metabolism. Some of these molecular functions have previously been associated with coral thermal responses. Protein kinase binding and protein serine/threonine kinase activity (GO:0019901 and GO:0004674) are known to be involved in early coral response to thermal and UV stress, shown in laboratory experiments to repress stress‐induced reactive oxygen species (Courtial et al. 2017). We detected enrichment of the enzyme glutathione S‐transferase omega‐1 (EC 2.5.1.18; GO:0045174), which is upregulated in some coral species following long‐term (60 days) heat stress exposure (Dias et al. 2019) and plays a key role in coral cellular antioxidant and immune defence responses (Morrow et al. 2012). Last, the GO enrichment analysis revealed significant chaperone binding activity (GO:0051087) and proteasome binding activity (GO:0070628). Six of the 13 associated genes were annotated as Sacsin (DNAJC29), which is a co‐chaperone for the heat‐shock protein Hsp70 (Ménade et al. 2018; Parfitt et al. 2009) and is significantly up‐regulated when *Acropora* and *Pocillopora* species are exposed to high temperatures in lab conditions (Cunning et al. 2018; Cunning and Baker 2014; Hemond et al. 2014; Mayfield et al. 2018). Association studies in *Acropora millepora* have also detected Sacsin as putatively under balancing selection (Fuller et al. 2020), while other chaperone binding proteins have been associated with bleaching alert frequency (Selmoni et al. 2021).

Twenty molecular functions were significantly enriched for *P. damicornis* , associated with protein quality control, epigenetic regulation, membrane transport, lipid metabolism, signalling, adhesion and energy metabolism. These GO terms appear to represent cellular response to maintain proteostasis, membrane stability, and regulatory flexibility under thermal stress, where previous studies on *Pocillopora* spp. under environmental stress have similarly identified candidate genes involved in immunity, cellular homeostasis, metabolism and signalling (Lundgren et al. 2013; Mayfield et al. 2018; Poquita‐Du et al. 2024; Selmoni et al. 2021).

This study provides a non‐exhaustive investigation into molecular signatures of local adaptation, where our use of DArT‐seq technology in genotyping likely missed genomic regions harbouring adaptive loci owing to low genome coverage (Lowry et al. 2017). Our post‐filtering sequencing coverage resulted in one SNP per ~55 kbp for *A. muricata* and ~25 kpb for *P. damicornis* , while the average LD decay is estimated at around 15 kbp for the related species *A. millepora* (Fuller et al. 2020). Despite sparse genome coverage, RADseq methods remain powerful and efficient approaches to provide a random sampling of genome‐wide SNPs, particularly for non‐model organisms (reviewed in Catchen et al. 2017; Razgour et al. 2018). Although high coverage whole genome sequencing data would be optimal to comprehensively characterise the genetic architecture of adaptation (Barratt et al. 2024; Lowry et al. 2017), our study goals were to investigate population structure and test for evidence of thermal adaptive differentiation across WIO reefs, for which RADseq remains a useful tool (Catchen et al. 2017). As the genetic architecture of coral heat adaptation is likely polygenic (reviewed in Selmoni et al. 2024), RADseq methods are expected to capture at least some loci linked to heat adapted genes, facilitating investigations into putatively enriched gene functions associated with thermal stress and mapping the geographic distributions of heat adapted genotypes.

We stress caution in over‐interpreting results of GEA and GO term enrichment analyses. These models can only generate hypotheses to facilitate understandings of environmental influence on genotypes (Lasky et al. 2023; Lotterhos 2023; Selmoni et al. 2024). Indeed, the aforementioned limitations of RADseq methods mean that detected outliers remain indications of potentially interesting genomic regions under thermal adaptation and are unlikely to be the actual loci under selection. Future studies into thermal adaptation will require higher resolution whole genome genotyping, where candidate genes should be treated as preliminary results requiring experimental and/or in‐field validations (reviewed in Selmoni et al. 2024).

Numerous analytical factors can further affect GEA results. Here, we traded GEA power with reduced false positive rates when we corrected for regional population structure, such that molecular signatures of thermal adaptation that are also correlated with structure will not be detected here (Forester et al. 2018; Lotterhos 2023). GEAs also have reduced power to detect quantitative trait nucleotides (Lotterhos 2023). Because polygenic variation is expected to drive coral thermal adaptation (Rose et al. 2018), important genomic regions under selection are likely missed. Additional methods, such as genome‐wide association studies (GWAS) or quantitative trait locus (QTL) mapping, could help strengthen conclusions drawn from seascape genomic studies (Lasky et al. 2023; Lotterhos 2023), as could the inclusion of other genomic variants that are expected to play a major role in facilitating adaptation (e.g., structural variants; Mérot et al. 2020; Pokrovac and Pezer 2022; Wellenreuther et al. 2019). Nevertheless, our results provide valuable hypotheses of genomic regions conserved across populations in response to thermal pressures across a discontinuous reef system, to help guide future coral research and assist in regional coral reef management.

### 4.5 Local‐Level Reef Management Is Recommended Across the Western Indian Ocean

Coral reef ecosystems across the WIO are vulnerable to collapse under projected climatic conditions (Obura et al. 2022). As local scale stressors can affect regional coral reef structures (Green et al. 1987), we echo recommendations for coordinated local management across the WIO to maintain overall coral reef health for both species (Obura et al. 2022; Stefanoudis et al. 2023). We find similar spatial distributions of putatively heat adapted genotypes for both species across the WIO region despite different life‐history strategies, where southern reefs are more likely to harbor heat‐adapted genotypes than northern reefs. It seems that large distances of open ocean between reefs of the WIO restrict genetic exchange across the network, with consequences for the dispersal of thermally adapted genotypes in the region. Indeed, simulations of coral propagule dispersal across the WIO estimate over 100 generational steps of separation (about 500 years) between the Mascarene Islands and the Seychelles (Vogt‐Vincent et al. 2024).

Understanding the source locations of thermally adapted corals and connectivity with other reefs in the region can help guide conservation planning (Bozec et al. 2025; Selmoni et al. 2024; Selmoni, Rochat, et al. 2020). Here, we used an Adaptive Index to estimate the spatial distribution of potentially thermal adapted individuals across the WIO. This index was derived by projecting the environmental conditions of naïve reefs into the multivariate genotype–environment space defined by sampled reefs, thereby identifying locations where allelic compositions are most consistent with thermal adaptive potentials. Reefs identified as likely to host corals with higher adaptive potentials could be prioritised for inclusion in marine protected areas (Abelson et al. 2016; De Clippele et al. 2023), while reefs with low adaptive potential might require more intervention, such as assisted gene flow from more adapted reefs (Baums 2008; Hagedorn et al. 2021; Van Oppen et al. 2015). The Mascarene Islands seem to harbour coral genotypes with higher thermal tolerance associated with historical exposure to elevated temperatures with large fluctuations. As the Mascarene Islands are located up‐current of Madagascar and the east African coast via the South Equatorial Current (Phillips et al. 2021; Vogt‐Vincent et al. 2024), these reefs may serve as important sources of thermal resilience for west WIO coral populations (Matz et al. 2020; Oury et al. 2024). In contrast, the Seychelles Archipelago appears to host fewer heat adapted corals, likely due to proximity to deep ocean upwelling that brings cooler and more consistent water temperatures (Shlesinger and van Woesik 2023). With their relative isolation, coral reefs of the Seychelles may require assisted gene flow, with propagules imported from heat adapted reefs, such as from east African reefs that have already experienced recurrent mass bleaching (Elma et al. 2023). However, fine‐scale habitat variation appears to be an important source of heat adapted genotypes for *P. damicornis* . Local variation may help preserve functionally relevant genetic diversity in the face of climate change, where populations exposed to hotter microclimates (e.g., lagoons and back reefs) could provide adaptive alleles to local populations (Richardson et al. 2014).

Here, we used two connectivity indices (ICI and OCI) to obtain a first indication of how sea currents might connect corals of *A. muricata* and *P. damicornis* across the WIO. These indices are intended as a first visual summary for understanding connectivity, limited by their use of *F*_ST_ to calculate sea distance thresholds (see Meirmans and Hedrick 2011; Whitlock and Mccauley 1999), where formal demographic modelling will be needed going forward. Nonetheless, insights obtained from these seascape genomic analyses can be integrated into management frameworks to be co‐developed with local stakeholders (Stefanoudis et al. 2023). Future work will need to include seascape genomic analyses across the larger WIO network prior to making more definitive conclusions and preparing management strategies.

### 4.6 Conclusions

Local thermal conditions seem to drive thermal tolerance across reefs of the Western Indian Ocean. We found strong molecular signals of heat stress adaptation in corals from thermally variable environments, such as at the Rodrigues and Mauritius Islands. The genetic variants potentially implicated in thermal adaptation were associated with genes coding for proteins previously identified as important in coral stress responses, notably the heat‐shock protein co‐chaperone, Sacsin. While regional patterns of population structure indicate that large sea distances and strong oceanographic barriers between WIO reefs may affect genetic exchange between regions, fine‐scale reef habitat variability may provide sources of adaptive alleles to local populations. These findings call for local‐level management of reefs within a framework of a larger regional management plan. Further research into phenotypic expressions of hypothesised target genes will need to be carried out to validate adaptive potentials of corals during stressed conditions. Insights from this research contribute to a growing understanding of coral adaptation to thermal stress for informing conservation strategies aimed at preserving thermally tolerant genotypes across the WIO in the face of ongoing climate change.

## Funding

This study was funded by the Adaptation Fund (AF), who financed the ‘Restoring Marine Ecosystem Services by Rehabilitating Coral Reefs to Meet a Changing Climate Future’ project (PIMS no. 5736), implemented by the United Nations Development Programme (UNDP) Mauritius and Seychelles, with the support of the Ministry of Agro‐Industry, Food Security, Blue Economy, and Fisheries, and in the Republic of Seychelles with the support of the Ministry of Agriculture, Climate Change and Environment.

## Conflicts of Interest

The authors declare no conflicts of interest.

## Supporting information


**Data S1:** eva70206‐sup‐0001‐DataS1.docx.

**Data S2:** eva70206‐sup‐0002‐DataS2.docx.

**Data S3:** eva70206‐sup‐0003‐DataS3.xlsx.

## Data Availability

Genotype files, sampling locations, environmental data and scripts used for these analyses are available on Dryad (DOI: https://doi.org/10.5061/dryad.931zcrjxv; Guillaume et al. 2026). Raw DArT sequence data are available in the NCBI BioProject database under accession number PRJNA1277000. Metadata for sampled and genotyped individuals can also be found on GEOME at https://n2t.net/ark:/21547/GQW2.
