# Supplementary material for: Coral Genetic Structure in the Western Indian Ocean Mirrors Ocean Circulation and Thermal Stress History
Source: Evol Appl. 2026 Feb 17;19(2):e70206. doi: 10.1111/eva.70206 (PMC12913226; doi:10.1111/eva.70206)
Supplement: Supplementary file 1 — Data S1: eva70206‐sup‐0001‐DataS1.docx. [file EVA-19-e70206-s001.docx]

Coral genetic structure in the Western Indian Ocean mirrors ocean circulation and thermal stress history

**Supplementary material**

**Suppl. Methods.** Selection of sample sites. *(available as a separate document)*

**Suppl. Fig. S1**. Workflow

**Suppl. Fig. S2**. Environmental variable correlations

**Suppl. Fig. S3.** Pruning of putatively cryptic individuals

**Suppl. Fig. S4.** Spatial distribution of clonal individuals

**Suppl. Fig. S5.** AMOVA histograms

**Suppl. Fig. S6.** Connectivity Indices distribution across WIO

**Suppl. Table S1.** Sample site location and sample size

**Suppl. Table S2.** Environmental variable descriptions

**Suppl. Table S3.** Species genetic ID

**Suppl. Table S4.** Genomic filtering

**Suppl. Table S5.** Pairwise F_ST_

**Suppl. Table S6.** SetRank analyses *(available as a separate document)*

**Suppl. Table S7.** Connectivity Indices for sampled sites

**Suppl. Fig. S1.** **Workflow.** Schema of the methods, highlighting the input data and analyses conducted, where we note where in the methods sections more details can be found.

**
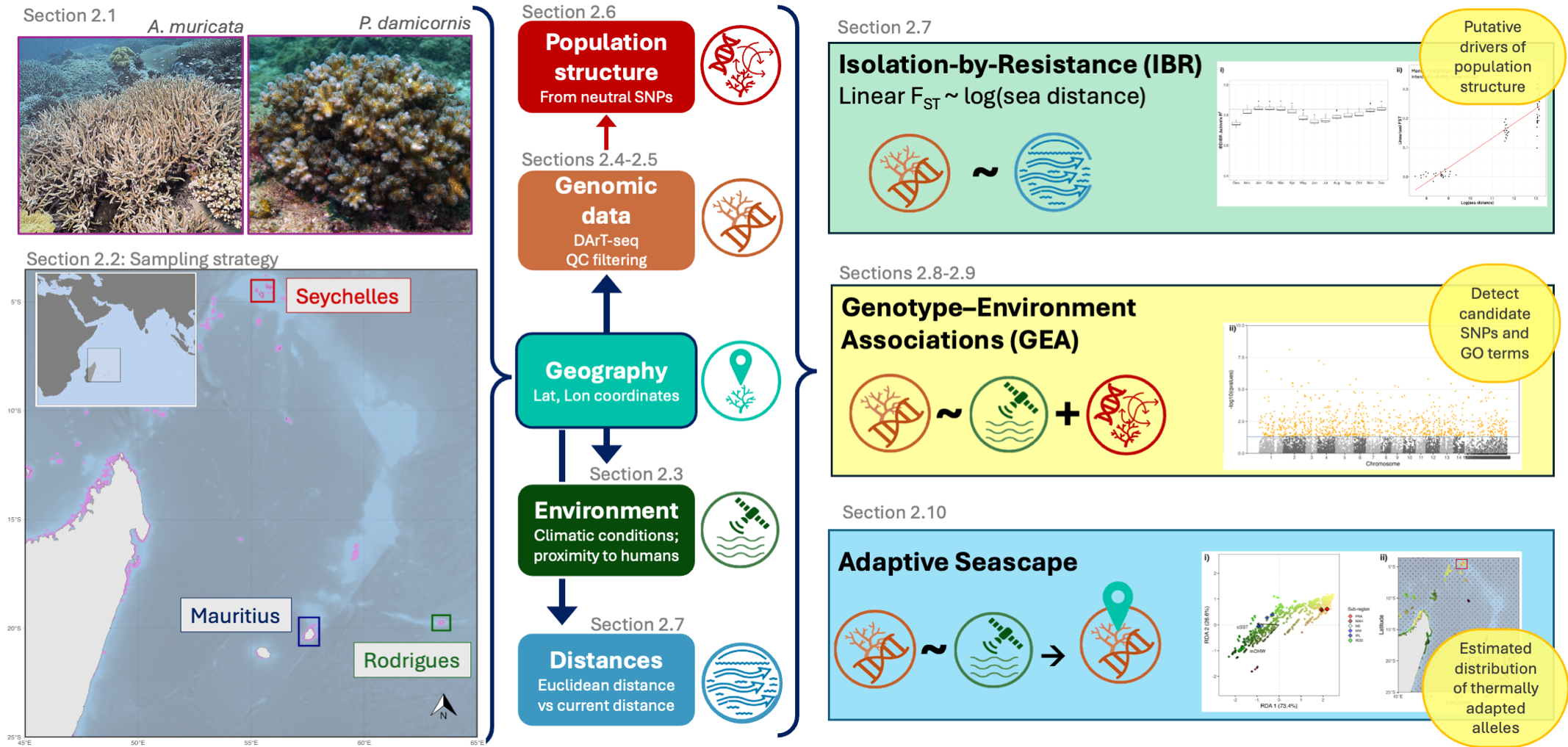
**

**Coral image credits:** (left) *A. muricata* taken in the Seychelles Islands by Charlie Veron; (right) *P. damicornis* taken in Brunei by Emre Turak and Lyndon DeVantier. Images obtained from Corals of the World website in 2025.

**Suppl. Fig. S2.** **Environmental variable correlations.** Correlation plots using Spearman non-parametric tests for (**a**) all 17 environmental variables considered and (**b**) the final 10 uncorrelated variables. See **Suppl. Table S1** for details on environmental variables and corresponding acronyms.

(**a**)


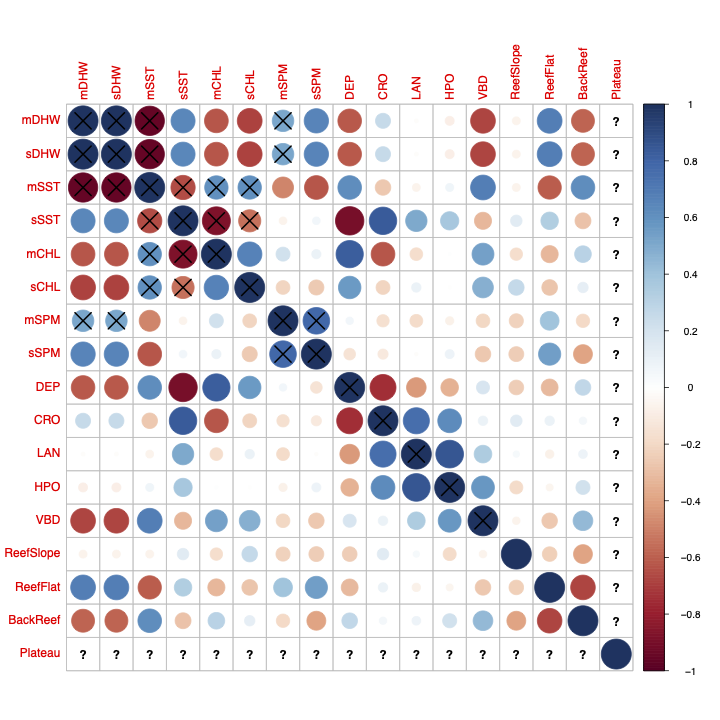


(**b**)

**
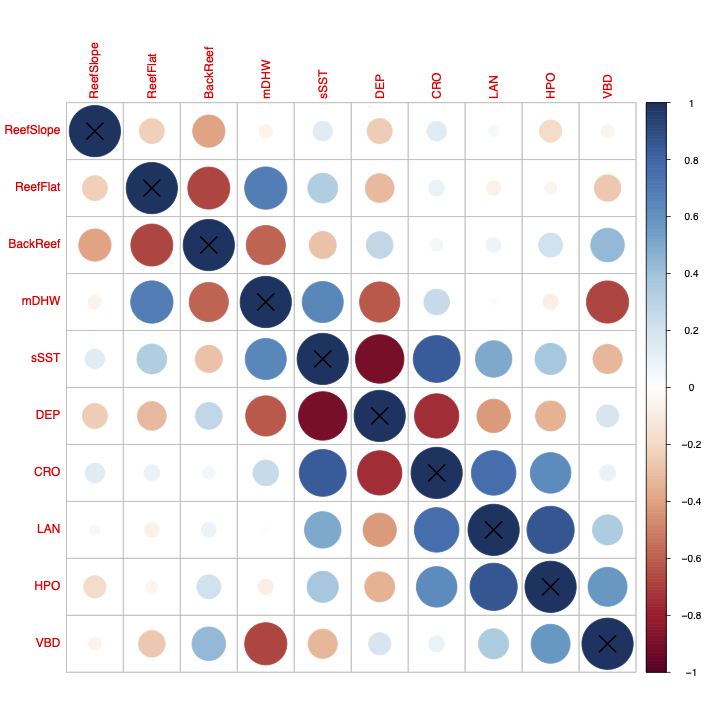
**

**Suppl. Fig. S3. Filtering of putatively cryptic individuals.** Analysis of principal coordinates (PCoA) and F_ST_ to identify putatively cryptic individuals sampled in the field for (**a**) *Acropora* *muricata* and (**b**) *Pocillopora damicornis*.

**(i)** Pre-filtering and **(ii)** post-filtering PCoA biplots for axes 1–4, indicating the distribution of individuals in relation to each other, coded by study sites (top row, coloured by study region and shaped by site) and by PCoA-based clustering (bottom row, colours reflect different groups). **(iii)** Maps highlighting the spatial distribution of genetic groups identified using PCoA at Seychelles (SEY), Mauritius (MAU), and Rodrigues (ROD). Each point represents a sampled individual, with colours matching the groupings of panel (**i**), with a jitter applied to facilitate investigation of genetic identification of individuals at the same site. **(iv)** Pre-filtering and **(v)** post-filtering pairwise F_ST_ values, represented as heatmaps for each PCoA group with low F_ST_ in dark green and high F_ST_ in dark red.

**(a) *Acropora muricata***

| **Pre-filtering** | **Post-filtering** | |
| --- | --- | --- |
| **i)**  **** | | **ii)**  **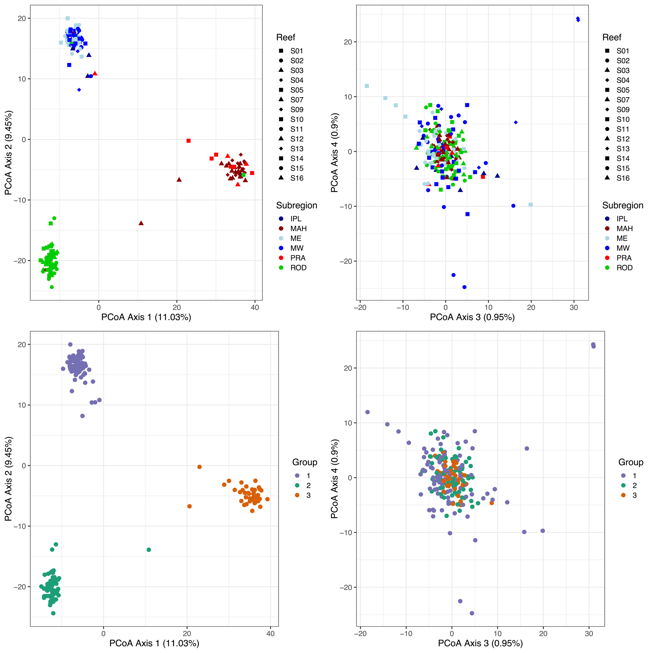** |
| **iii)**  **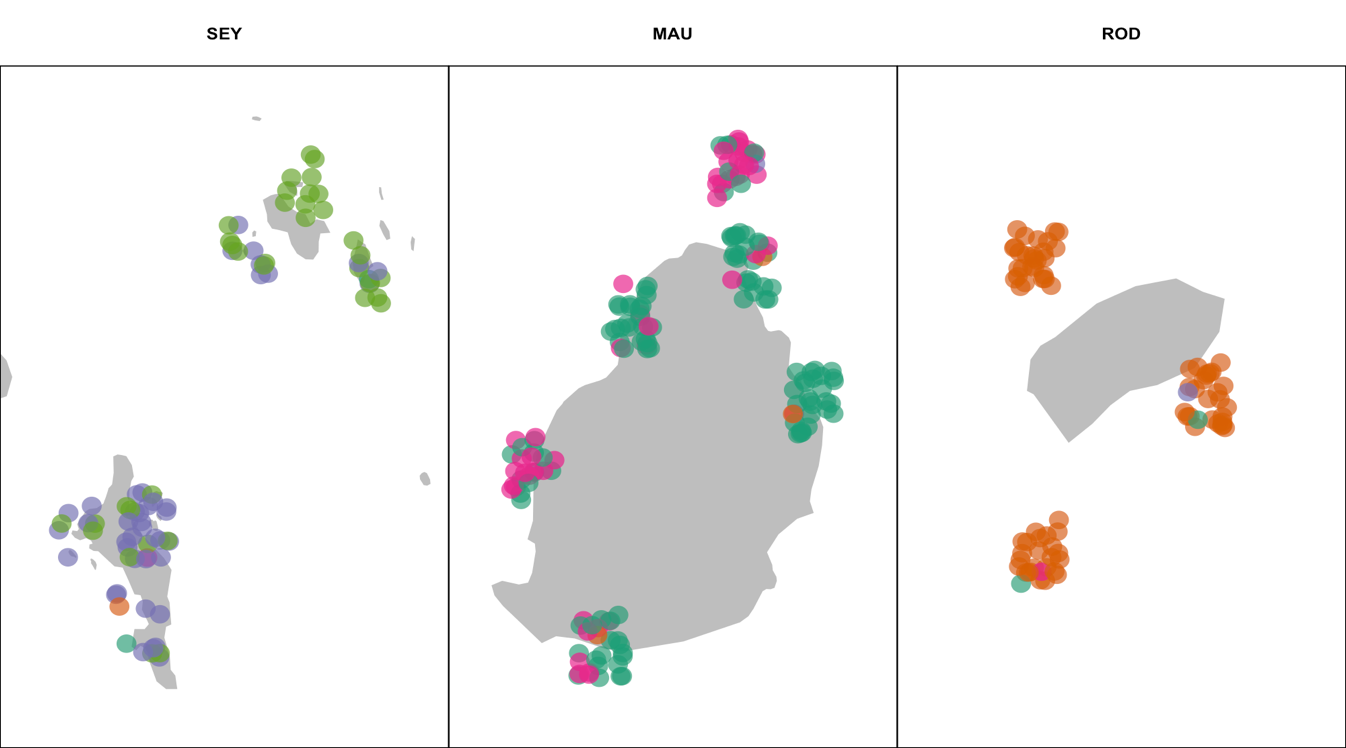** | | |
| **iv)**  **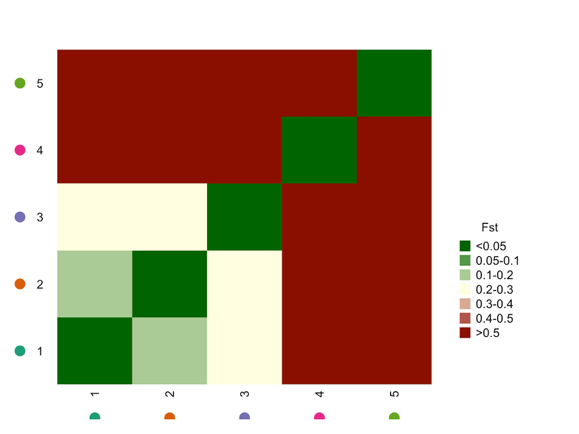** | **v)**  **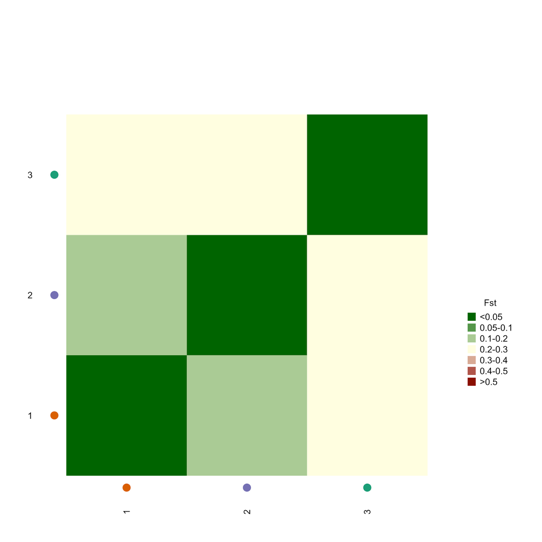** | |

**(b) *Pocillopora damicornis***

| **Pre-filtering** | **Post-filtering** | |
| --- | --- | --- |
| **i)**  **** | | **ii)**  **** |
| **iii)**  **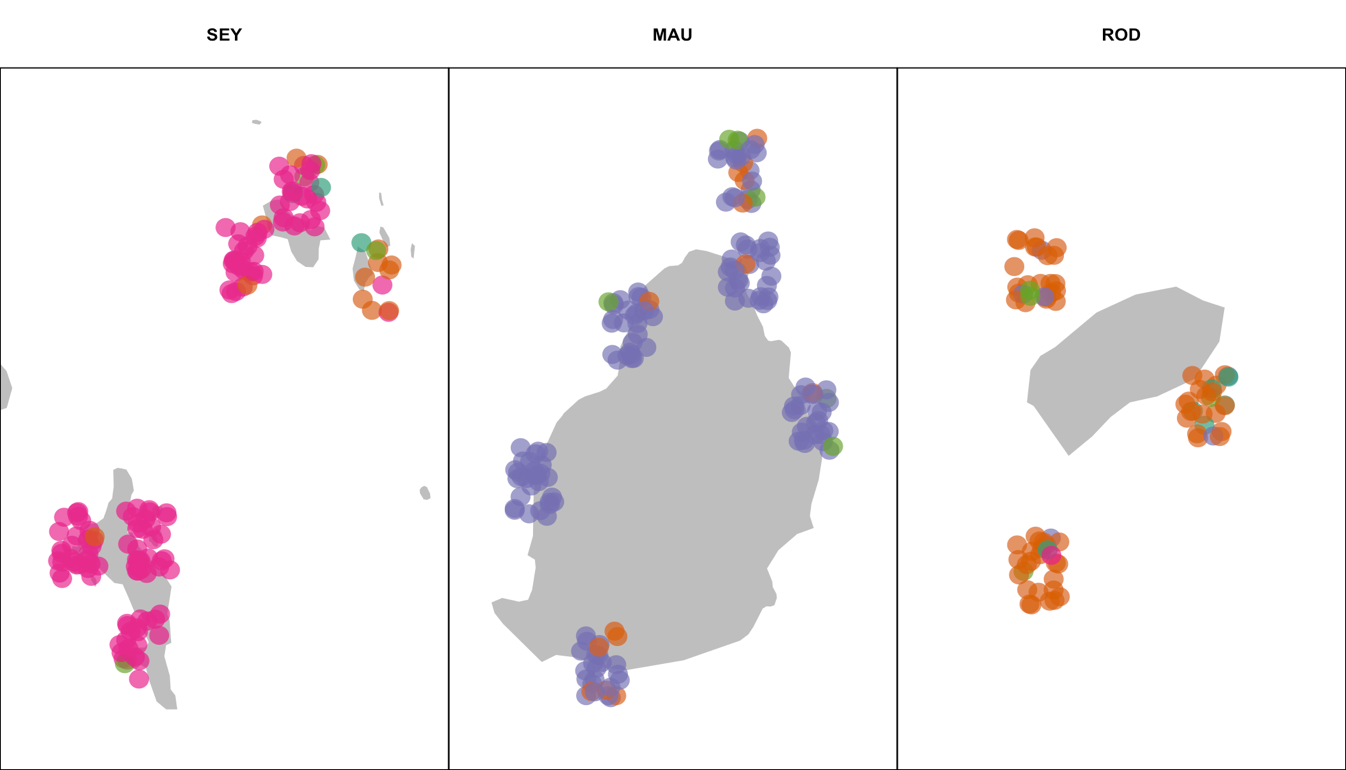** | | |
| **iv)**  **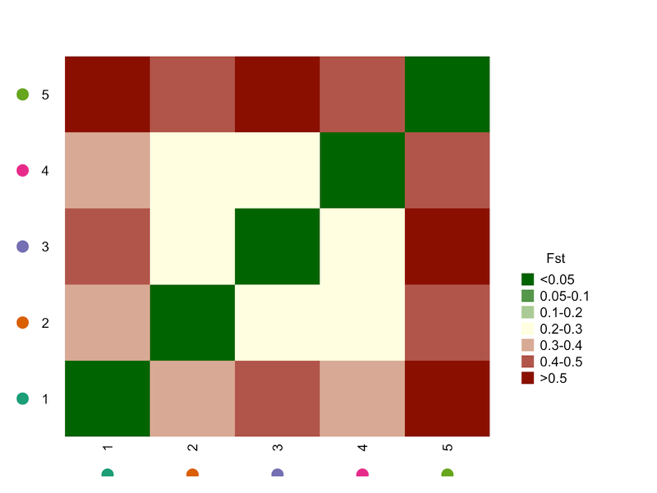** | **v)**  **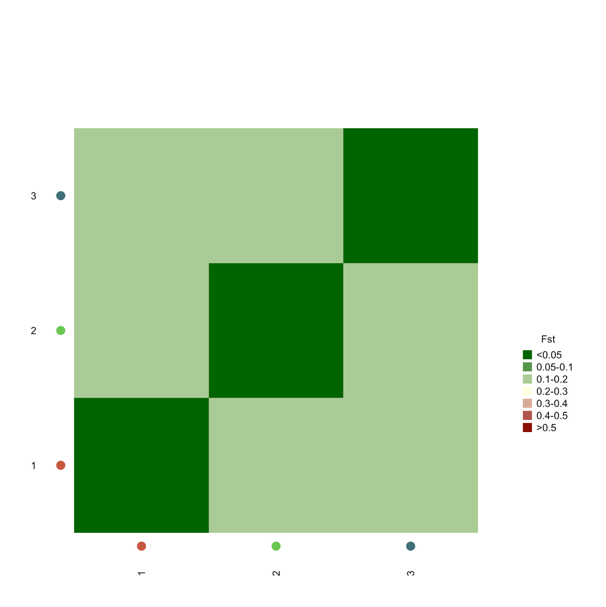** | |

**Suppl. Fig. S4. Filtering of clonal individuals.** Spatial distribution of clonal individuals across the Seychelles (SEY), Mauritius (MAU), and Rodrigues (ROD) sample sites for **(a)** *Acropora muricata* and **(b)** *Pocillopora damicornis*. Each point represents a sampled individual, where we defined clonal individuals as those sharing over 95% or 90% of their genotypes, respectively. **i)** Distribution of clonal individuals at each region, where points in blue are those not genetically similar with other individuals, and points in red are clones. Lines between points indicate clonal pairs. **ii)** Percentage of clones at each sample site, with point size representing sample size and colour representing % clones. Lines between points indicate connection between sampled clonal pairs. Note for each map, we applied a jitter to facilitate investigation of genetic identification of individuals at the same site.

**(a) *Acropora muricata***

**i)**

**
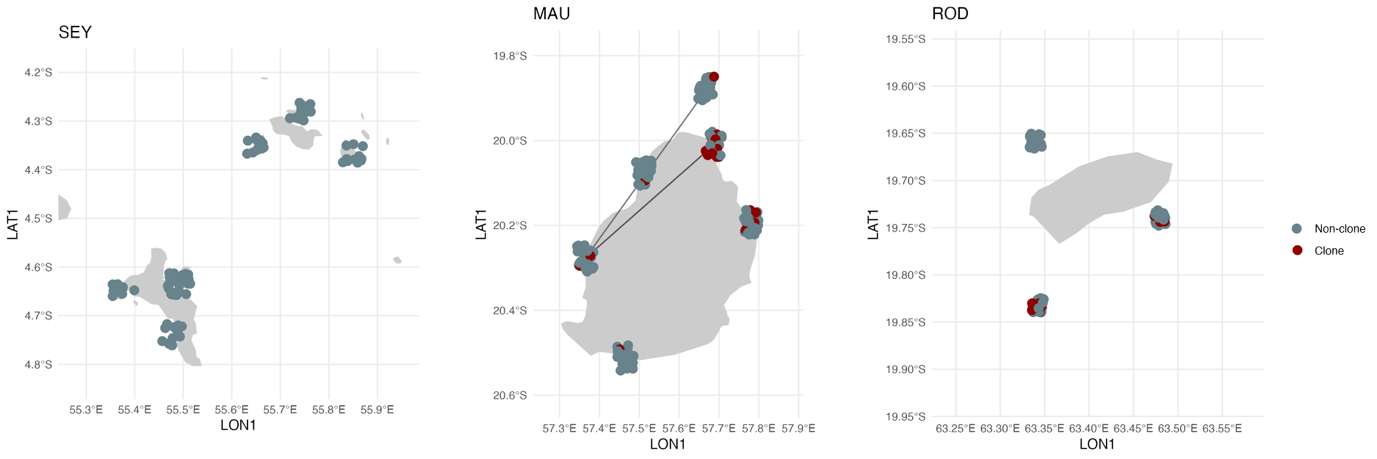
**

**ii)**

**
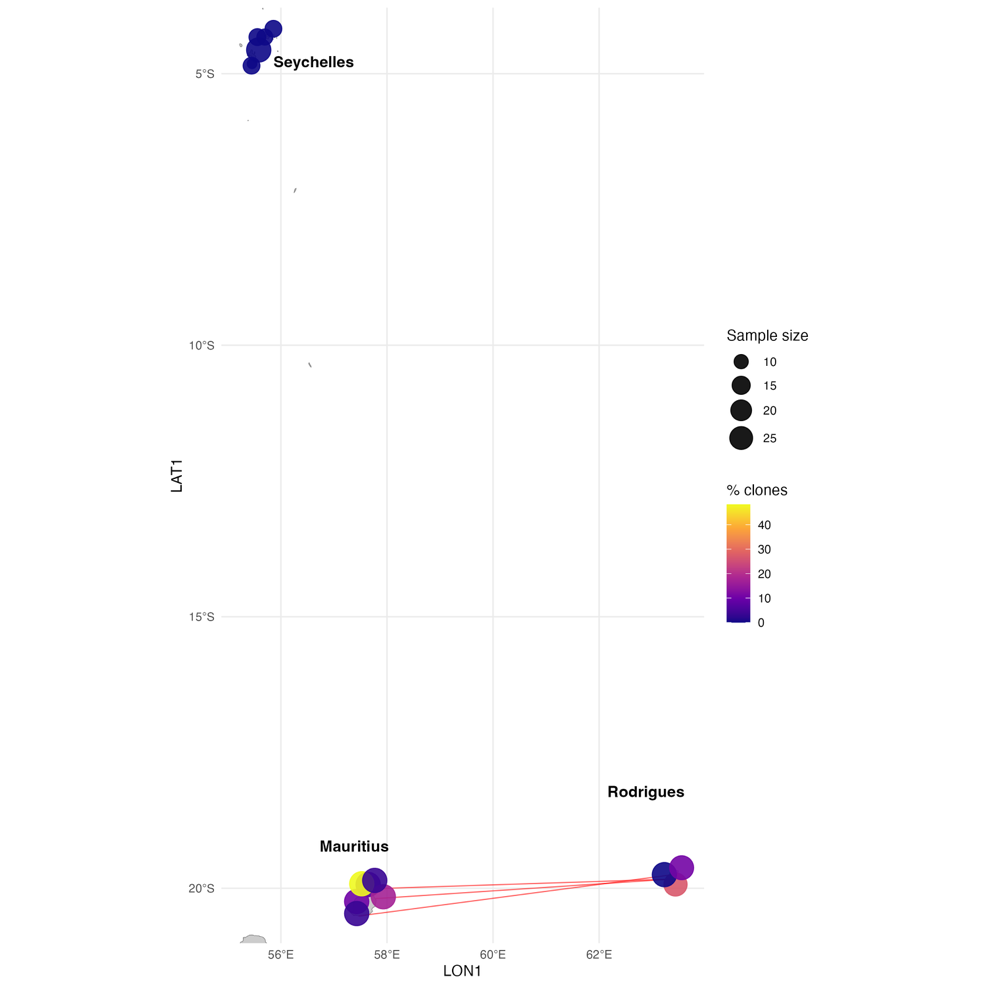
**

**(b) *Pocillopora damicornis***

**i)**

**
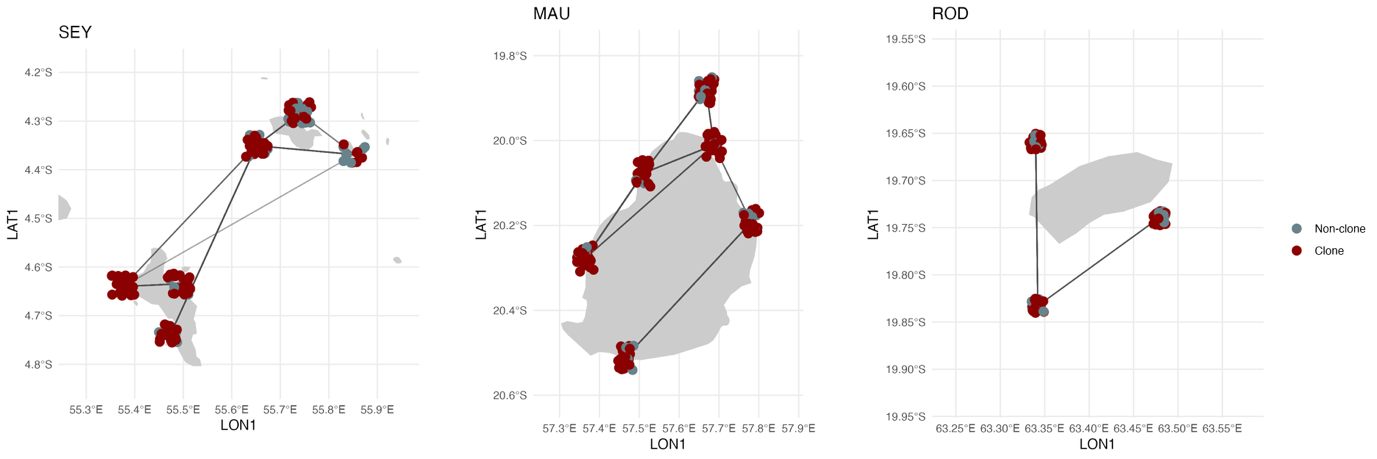
**

**ii)**

**
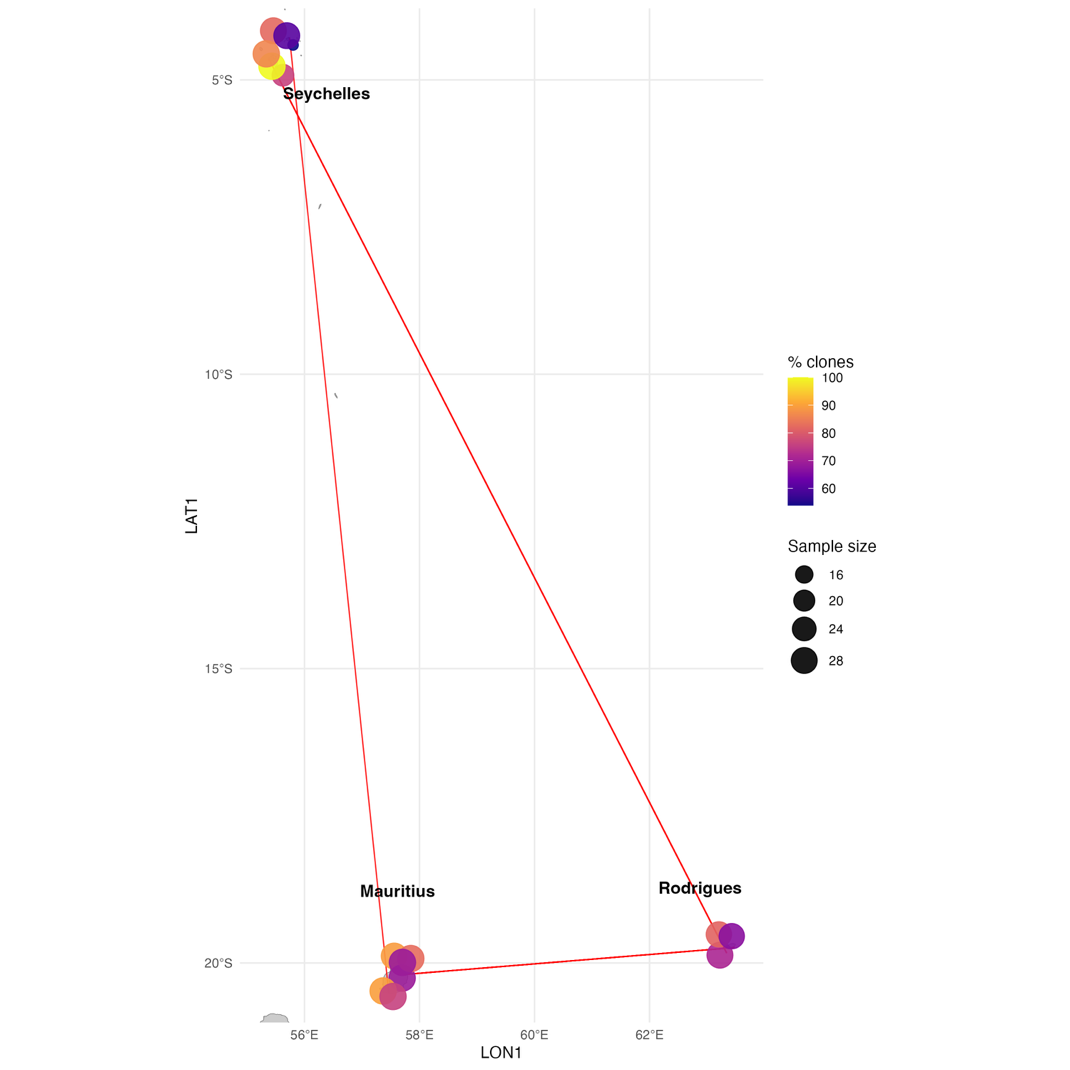
**

**Suppl. Fig. S5.** **AMOVA histograms.** Analysis of molecular variance (AMOVA) to detect population differentiation using SNP markers comparing allele frequency variation of SNPs for (**a**) *Acropora muricata* and (**b**) *Pocillopora damicornis*. Histograms represent the distribution of randomly permutated values (grey boxes) compared to the observed results (black line) for SNP allele frequency variation within samples, between samples, between sample sites, between subregions, and between regions.

| **(a) *Acropora muricata***   |
| --- |
| **(b) *Pocillopora damicornis***  **** |

**Suppl. Fig. S6.** **Inbound (ICI) and Outbound (OCI) Connectivity Indices** for (**a**) *Acropora muricata* and (**b**) *Pocillopora damicornis* for 15 sampled reefs across the Western Indian Ocean. Inbound and Outbound Connectivity indices indicate the respective area (in km^2^) of reefs either up or down-current of a target reef within a sea distance corresponding to genetic connectivity thresholds. Species-specific F_ST_ value thresholds were set as the average of the regional maximum F_ST_ values to capture average genetic differentiation for reef regions, with *A. muricata:* F_ST_≤0.014 and *P damicornis:* F_ST_≤0.067.

**(a) *Acropora muricata***

**
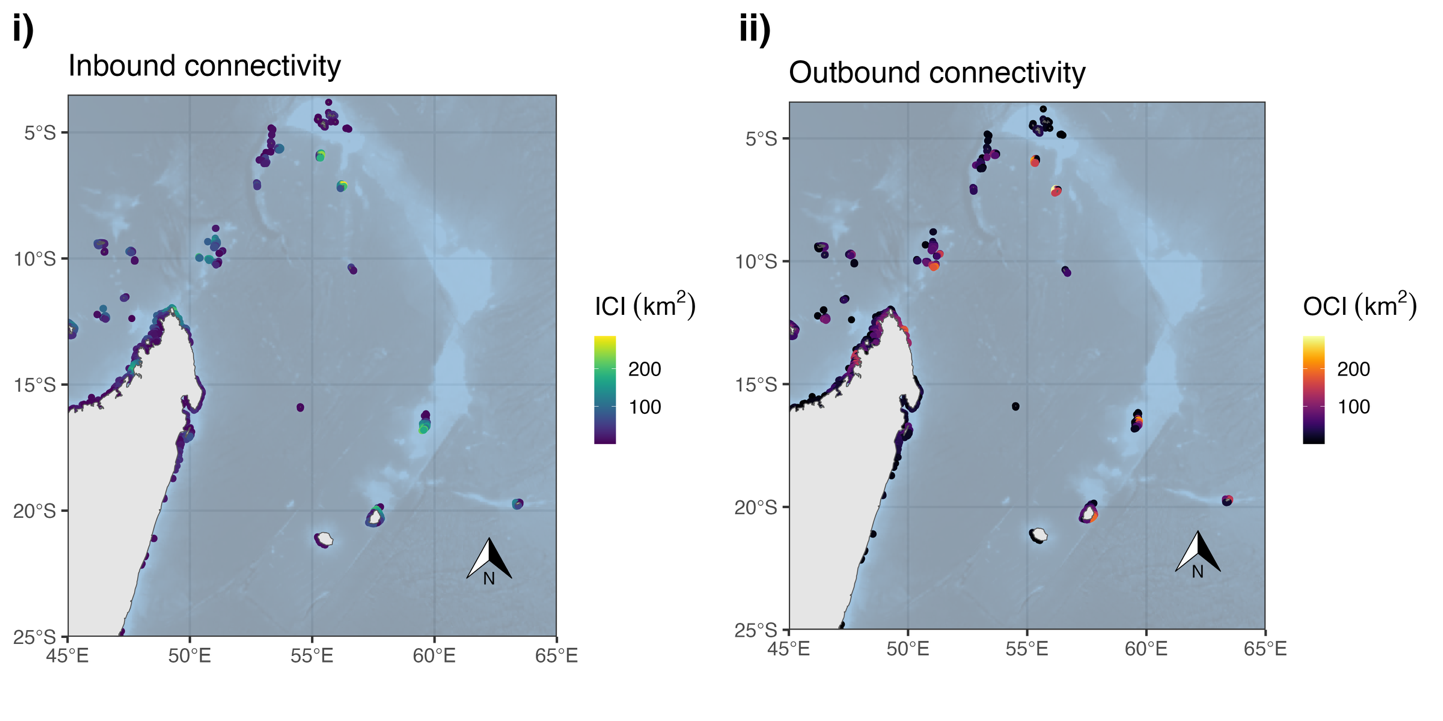
**

**(b) *Pocillopora damicornis***

**
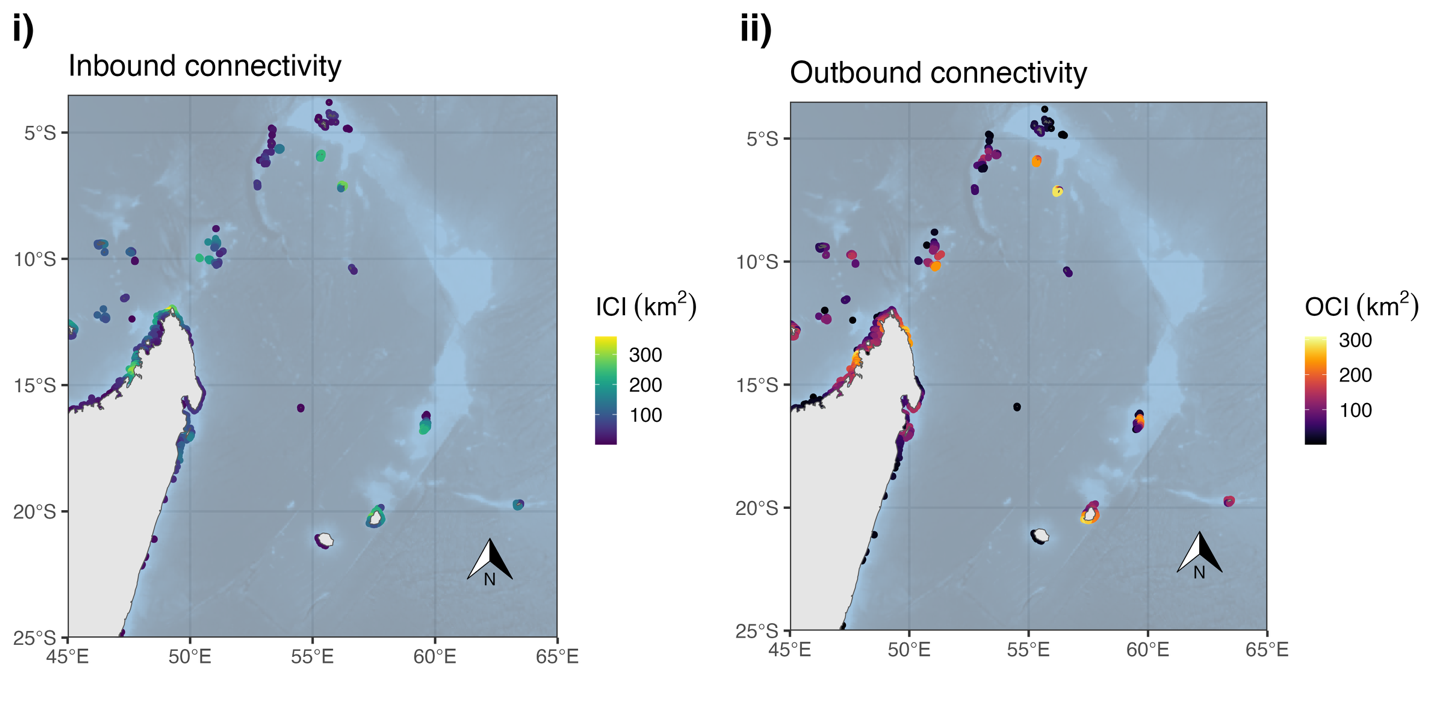
**

**Suppl. Table S1.** **Sample sites and sample sizes.** Summary of the 15 sample sites where *Acropora muricata* and *Pocillopora damicornis* colonies were collected for genotyping in the Western Indian Ocean. Summarised in the table: Region, Sub region, reef name, average latitude (LAT) and longitude (LON), diving depth in meters (Depth), and the number of colonies remaining after SNP genotype filtering.

Region abbreviations: MAU = Maurice, ROD = Rodrigues, SEY = Seychelles. Sub-region abbreviations: IPL = Ile Plate, ME = East Mauritius, MW = West Mauritius, ROD = Rodrigues, MAH = Mahé, PRA = Praslin.

| **Code** | **Region** | **Sub-region** | **Reef name** | **LAT** | **LON** | **Depth (m)** | **Number of *A. muricata*** | | **Number of *P. damicornis*** | |
| --- | --- | --- | --- | --- | --- | --- | --- | --- | --- | --- |
|  |  |  |  |  |  |  | Sampled | Post-filtering | Sampled | Post-filtering |
| S12 | MAU | IPL | Ile Plate | -19.881 | 57.668 | 1-3 | 29 | 7 | 29 | 2 |
| S02 | MAU | ME | Belle Mare | -20.192 | 57.781 | 1-3 | 29 | 25 | 29 | 9 |
| S10 | MAU | ME | Gd Bourg Melville | -20.010 | 57.687 | 1-3 | 29 | 15 | 29 | 10 |
| S11 | MAU | MW | Balaclava | -20.077 | 57.510 | 1-3 | 29 | 23 | 28 | 3 |
| S13 | MAU | MW | FlicFlac | -20.277 | 57.365 | 1-3 | 29 | 8 | 29 | 9 |
| S01 | MAU | MW | St Felix | -20.512 | 57.465 | 1-3 | 29 | 19 | 29 | 2 |
| S16 | ROD | ROD | 80 Brisants | -19.833 | 63.342 | 1-2 | 26 | 18 | 28 | 9 |
| S15 | ROD | ROD | Gd Bassin Bretagne | -19.659 | 63.340 | 1-2 | 29 | 25 | 27 | 7 |
| S14 | ROD | ROD | SEMPA | -19.739 | 63.479 | 1-2 | 28 | 27 | 27 | 7 |
| S09 | SEY | MAH | Anse La Mouche | -4.739 | 55.474 | 1-2 | 13 | 10 | 21 | 3 |
| S04 | SEY | MAH | Baie Ternay | -4.640 | 55.377 | 1-2 | 8 | 5 | 29 | 5 |
| S03 | SEY | MAH | Ile Au Cerf | -4.635 | 55.491 | 1-4 | 29 | 19 | 29 | 10 |
| S07 | SEY | PRA | Coco | -4.369 | 55.853 | 3-10 | 13 | 4 | 13 | 0 |
| S05 | SEY | PRA | Cousine | -4.351 | 55.651 | 3-6 | 12 | 6 | 28 | 9 |
| S06 | SEY | PRA | Curieuse | -4.284 | 55.740 | 3-7 | 13 | 0 | 28 | 12 |
|  |  |  |  |  |  | **Total** | **345** | **211** | **403** | **117** |

**Suppl. Table S2.** **Environmental variable description.** Variables used to characterise the reefs around the study regions of Mauritius, Rodrigues and the Seychelles in the Western Indian Ocean. Provided are: acronym used in the main text, full variable name, calculated spatial resolution, calculated temporal window, open-access website from which variables are sourced (RECIFS: Selmoni et al., 2020, or the Allen Coral Atlas (ACA): Allen Coral Atlas 2022), details of the variable, and the origin of the raw data used to make the variables. The 10 variables in bold and with an asterisk (*) were used to characterise the reefs and in GEAs.

| **Acronym** | **Variable** | **Spatial resolution** | **Temporal window** | **Repository** | **Details** | **Source** |
| --- | --- | --- | --- | --- | --- | --- |
| ***DEP** | Depth | 5x5km | n/a | RECIFS | Average depth around reef calculated with a 5km buffer | Global Multi-Resolution Topography Data Synthesis |
| ***mDHW** | Degree Heating Week (mean) | 5x5km | Monthly max from 1985-2021 | RECIFS | Overall mean of the monthly maxima DHW (accumulated thermal stress over previous 12 weeks | NOAA Coral Reef Watch |
| sDHW | Degree Heating Week (sd) | 5x5km | Monthly max from 1985-2021 | RECIFS | Overall sd of the monthly maxima DHW (accumulated thermal stress over previous 12 weeks | NOAA Coral Reef Watch |
| mSST | Sea Surface Temperature (mean) | 5x5km | Monthly ave from 1985-2021 | RECIFS | Overall mean of the monthly SST | NOAA Coral Reef Watch |
| ***sSST** | Sea Surface Temperature (sd) | 5x5km | Monthly ave from 1985-2021 | RECIFS | Overall sd of the monthly SST | NOAA Coral Reef Watch |
| mCHL | Chlorophyll concentration (mean) | 5x5km | Monthly ave from 1997-2021 | RECIFS | Overall mean of the monthly average of mass chlorophyll-a concentration in seawater | Copernicus Marine Service |
| sCHL | Chlorophyll concentration (sd) | 5x5km | Monthly ave from 1997-2021 | RECIFS | Overall sd of the monthly average of mass chlorophyll-a concentration in seawater | Copernicus Marine Service |
| mSPM | Suspended Particulate Matter (mean) | 5x5km | Monthly ave from 1997-2021 | RECIFS | Overall mean of the mass concentration of suspended matter in seawater | Copernicus Marine Service |
| sSPM | Suspended Particulate Matter (sd) | 5x5km | Monthly ave from 1997-2021 | RECIFS | Overall sd of the mass concentration of suspended matter in seawater | Copernicus Marine Service |
| **Acronym** | **Variable** | **Spatial Res** | **Temporal window** | **Source** | **Details** | **Origin of raw data** |
| ***CROP** | Density of cropland | 5x5km | Measured in 2015–2019 | RECIFS | Percentage of pixels in 5km buffer that correspond to cropland cover | Copernicus Global Land Service |
| ***LAND** | Density of land surface | 5x5km | n/a | RECIFS | Percentage of pixels in 5km buffer that correspond to land cover | Global Multi-Resoulution Topography Data Synthesis |
| ***HPOP** | Human population along coastline | 5x5km | Measured in 2000, 2005, 2010, 2015, 2020 | RECIFS | Mean human population density in the 5km buffer around the reef of interest | Center for International Earth Science Information |
| ***BOAT** | Boat detection | 5x5km | Measured in 2017–2021 | RECIFS | Mean of the percentage of boats detected using satellite imagery | Earth Observation Group, Payne Institute for Public Policy |
| ***ReefSlope** | Prop. reef slope 250m around reef | 10m | n/a | ACA | Submerged, sloping area extending seaward from the Reef Crest (or Flat) towards the shelf break. | Planet Dove satellite imagery |
| ***ReefFlat** | Prop. reef flat 250m around reef | 10m | n/a | ACA | Adjacent to the seaward edge of the reef, Outer Reef Flat is a levelled (near horizontal) broad and shallow carbonate platform, displaying distinct wave-driven zonation. | Planet Dove satellite imagery |
| ***BackReef** | Prop. back reef 250m around reef | 10m | n/a | ACA | A complex, interior - often gently sloping - reef zone occurring behind the Reef Flat. Of variable depth (but deeper than Reef Flat and more sloped), it is sheltered, sediment-dominated and often punctuated by coral outcrops. | Planet Dove satellite imagery |
| Plateau | Prop plateau 250m around reef | 10m | n/a | ACA | Deep submerged (> 5 m), hard-bottomed, horizontal to gently sloping (angle shallower than 10 ° approx), seaward facing reef platform. | Planet Dove satellite imagery |

**Suppl. Table S3. Species ID** **Genetic confirmation.** Genetic confirmation of retained *Pocillopora* samples (i.e., after filtering for clones and cryptic individuals) as *P. damicornis,* using DArT sequences within the 18S and ITS-2 ribosomal DNA (rDNA) regions. Five individuals from each of the four *Pocillopora* PCoA genetic groups (Suppl. Fig. S3B) were randomly selected for analysis. These 20 individuals were queried against rDNA sequences obtained from NCBI (Genbank ID) for four target *Pocillopora* species (*P. damicornis*, P*. verrucosa, P. eydouxi*, *P. elegans*) and two outgroup species (*Stylophora pistillata* and *Seriatopora* sp.). The Percentage of Identity indicates how much our 20 samples aligned to the target species for each queried sequence, alongside the length of alignment (base pairs; bp) and the expectation value (E-value) of the BLAST hits. As the sequences for all 20 individuals at these loci were 100% in alignment with each other, here we show results from the genotyped individual 3586603 (ID ‘01_P_12’ from Site S01 in Mauritius). Nucleotide alignments for the 18S and ITS-2 sequences of the 20 individuals alongside three cryptic individuals and target species are shown below (aligned using ClustalW).

| **DNA region** | **Query sequence** | **Target sequence (Genbank)** | **Target species** | **Percentage of identity** | **Alignment length (Bp)** | **E-value** |
| --- | --- | --- | --- | --- | --- | --- |
| 18S | 3586603_18S | PQ434668.1 | *Pocillipora damicornis* | 100.00 | 105 | 1.42e^-54^ |
| 18S | 3586603_18S | XR_010716799.1 | *Pocillopora verrucosa* | 98.53 | 67 | 8.76e^-32^ |
| 18S | 3586603_18S | LT631145.1 | *Stylophora pistillata* | 95.24 | 105 | 3.09e^-46^ |
| 18S | 3586603_18S | LT631140.1 | *Seriatopora* sp. | 95.24 | 105 | 3.09e^-46^ |
| ITS-2 | 3586603_ITS2 | KF846519.1 | *Pocillopora damicornis* | 99.06 | 108 | 5.25e^-54^ |
| ITS-2 | 3586603_ITS2 | HM013854.1 | *Pocillopora eydouxi* | 96.26 | 108 | 5.29e^-49^ |
| ITS-2 | 3586603_ITS2 | EU314802.1 | *Pocillopora elegans* | 96.26 | 108 | 5.29e^-49^ |
| ITS-2 | 3586603_ITS2 | OK448746.1 | *Pocillopora verrucosa* | 94.29 | 36 | 2.59e^-12^ |

18S rDNA Alignment


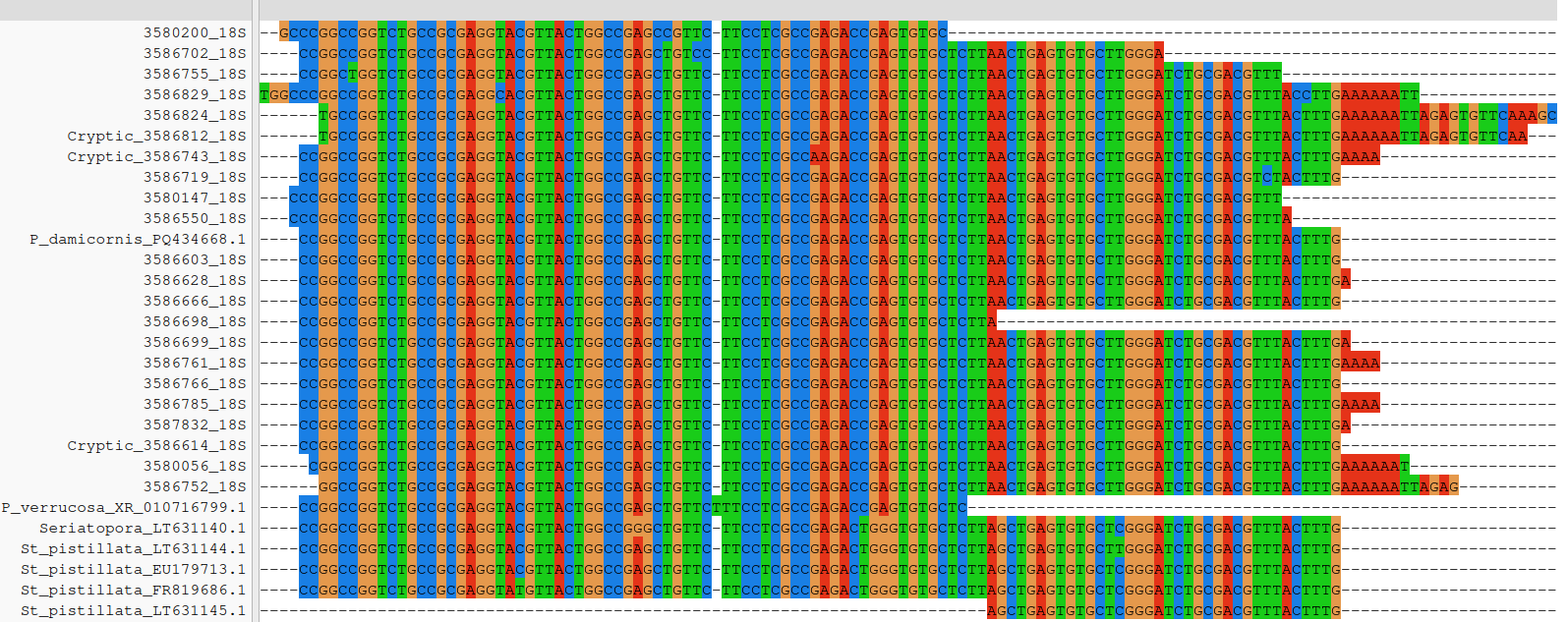


ITS-2 rDNA alignment


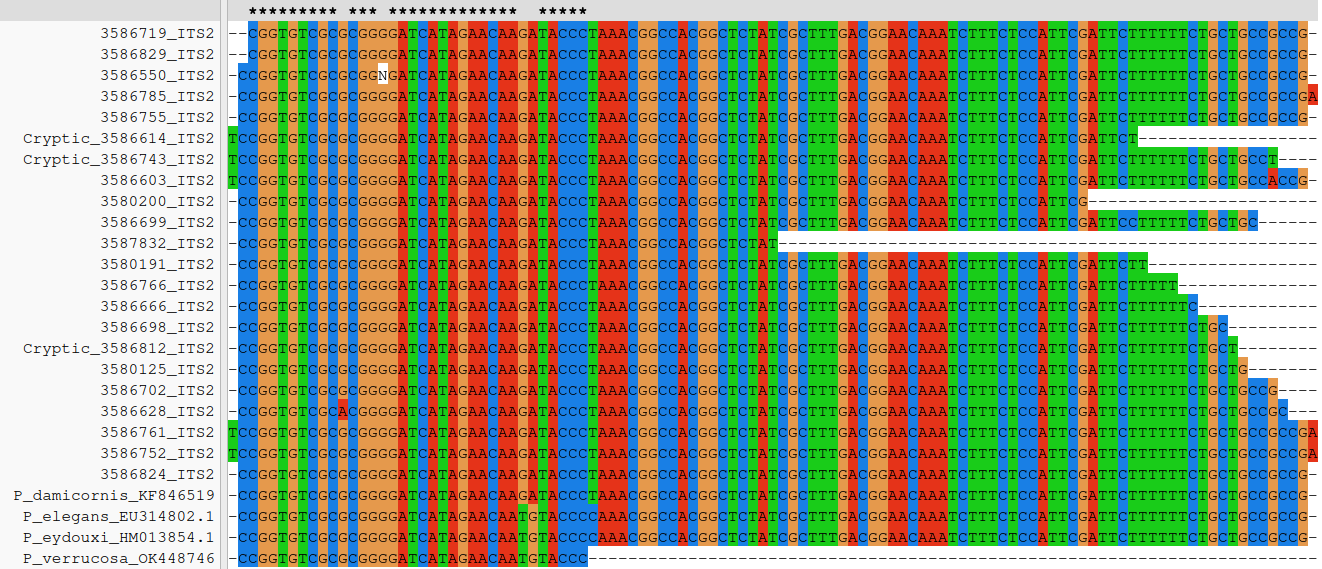


**Suppl. Table S4.** **Genomic filtering.** Number of SNPs and individuals retained after each step of genomic filtering for *Acropora muricata* and *Pocillopora damicornis* at all sampled reefs of the WIO. The first row indicates the number of SNPs and individuals in the raw DArT-seq genetic file, followed by numbers retained after running a BLAST against the reference genomes. We then perform a soft filtering (loci and individuals each with 50% missingness) to identify and remove putatively cryptic individuals from the initial DART file. For *P. damicornis* we perform a second filtering for cryptic individuals, using a hard filtering step (loci and individuals each with 80% missingness). Finally, we perform a hard filtering step on the initial DART file that has been pruned for both cryptic and clonal individuals, using 80% missingness threshold for each of loci and individuals, with a MAF <5%. We also show values following analysis of LD between loci (shaded out row), where we decided to retain all SNPs for the downstream analyses. Finally, we show the number of SNPs detected as outlier Loci using *PCAdapt* and the number of SNPs retained for the neutral dataset.

| **Filtering step** | ***Acropora muricata*** | | ***Pocillopora damicornis*** | |
| --- | --- | --- | --- | --- |
|  | **SNPs** | **Individuals** | **SNPs** | **Individuals** |
| **DArT-seq** | 73,253 | 345 | 65,708 | 403 |
| **BLAST** | 63,511 | 345 | 52,219 | 403 |
| **MNsnp 50%; MNind 50%** | 38,666 | 330 | 44,327 | 396 |
| **Cryptic removal #1** | 63,511 | 257 | 52,219 | 380 |
| **MNsnp 80%; MNind 80%** | 14,530 | 230 | 22,656 | 369 |
| **Clonal removal** | 63,511 | 235 | 52,219 | 128 |
| **MNsnp 50%; MNind 50%** | N/a | N/a | 31,179 | 121 |
| **Cryptic removal #2** | N/a | N/a | 52,219 | 108 |
| **MNsnp 80%; MNind 80%** | 14,755 | 211 | 19,146 | 97 |
| **MAF: Final** | 7,663 | 211 | 13,190 | 97 |
| **LD pruning** | 4,002 | 211 | 6,838 | 97 |
| **Outlier loci** *PCAdapt* | 124 | 211 | 103 | 97 |
| **Neutral loci** | 7,539 | 211 | 13,087 | 97 |

**Suppl. Table S5. Pairwise F_ST_** between study sites for *A. muricata* (lower triangle) and *P. damicornis* (upper triangle) based on neutral SNP genotype matrices. Sites are grouped and coloured by region and subregion. F_ST_ ≥0.20 are highlighted in yellow.

|  |  |  |  | ***Pocillopora damicornis*** | | | | | | | | | | | | | | | |
| --- | --- | --- | --- | --- | --- | --- | --- | --- | --- | --- | --- | --- | --- | --- | --- | --- | --- | --- | --- |
|  |  |  |  | **Seychelles** | | | | | | **Mauritius** | | | | | | **Rodrigues** | | | |
|  |  |  |  | PRA | | | MAH | | | IPL | MW | | | ME | | ROD | | | |
|  |  |  |  | S06 | S05 | S07 | S03 | S04 | S09 | S12 | S01 | S11 | S13 | S02 | S10 | S14 | S15 | S16 |  |
| ***Acropora muricata*** | **Seychelles** | PRA | S06 |  | 0.02 | – | 0.04 | 0.04 | 0.04 | 0.18 | 0.13 | 0.13 | 0.18 | 0.18 | 0.19 | 0.14 | 0.16 | 0.18 |  |
|  |  |  | S05 | – |  | – | 0.04 | 0.04 | 0.08 | 0.21 | 0.17 | 0.17 | 0.21 | 0.21 | 0.22 | 0.16 | 0.18 | 0.20 |  |
|  |  |  | S07 | – | 0.00 |  | – | – | – | – | – | – | – | – | – | – | – | – |  |
|  |  | MAH | S03 | – | 0.00 | 0.01 |  | 0.07 | 0.09 | 0.23 | 0.19 | 0.18 | 0.22 | 0.22 | 0.23 | 0.18 | 0.20 | 0.22 |  |
|  |  |  | S04 | – | 0.00 | 0.01 | 0.00 |  | 0.09 | 0.18 | 0.12 | 0.13 | 0.19 | 0.20 | 0.21 | 0.10 | 0.12 | 0.14 |  |
|  |  |  | S09 | – | 0.00 | -0.02 | 0.00 | 0.00 |  | 0.22 | 0.17 | 0.16 | 0.20 | 0.20 | 0.22 | 0.14 | 0.17 | 0.20 |  |
|  | **Mauritius** | IPL | S12 | – | 0.12 | 0.09 | 0.15 | 0.16 | 0.09 |  | 0.05 | 0.03 | 0.03 | 0.06 | 0.04 | 0.09 | 0.11 | 0.19 |  |
|  |  | MW | S01 | – | 0.20 | 0.17 | 0.21 | 0.23 | 0.16 | 0.01 |  | -0.01 | 0.03 | 0.04 | 0.06 | 0.03 | 0.06 | 0.14 |  |
|  |  |  | S11 | – | 0.19 | 0.16 | 0.21 | 0.23 | 0.16 | 0.01 | 0.00 |  | -0.01 | 0.03 | 0.04 | 0.04 | 0.06 | 0.13 |  |
|  |  |  | S13 | – | 0.20 | 0.17 | 0.22 | 0.24 | 0.16 | 0.02 | 0.02 | 0.02 |  | 0.03 | 0.03 | 0.11 | 0.12 | 0.19 |  |
|  |  | ME | S02 | – | 0.19 | 0.17 | 0.21 | 0.23 | 0.16 | 0.01 | 0.00 | 0.00 | 0.02 |  | 0.02 | 0.11 | 0.14 | 0.21 |  |
|  |  |  | S10 | – | 0.20 | 0.18 | 0.21 | 0.24 | 0.16 | 0.01 | 0.01 | 0.01 | 0.02 | 0.01 |  | 0.13 | 0.14 | 0.21 |  |
|  | **Rodrigues** | ROD | S14 | – | 0.20 | 0.19 | 0.21 | 0.24 | 0.16 | 0.11 | 0.13 | 0.13 | 0.14 | 0.12 | 0.13 |  | 0.00 | 0.00 |  |
|  |  |  | S15 | – | 0.23 | 0.22 | 0.23 | 0.27 | 0.19 | 0.14 | 0.15 | 0.15 | 0.16 | 0.14 | 0.16 | 0.00 |  | 0.04 |  |
|  |  |  | S16 | – | 0.22 | 0.20 | 0.22 | 0.25 | 0.17 | 0.12 | 0.13 | 0.13 | 0.14 | 0.12 | 0.14 | 0.00 | 0.00 |  |  |

Sub-region abbreviations: PRA = Praslin, MAH = Mahé, IPL = Ile Plate, MW = West Mauritius, ME = East Mauritius, ROD = Rodrigue

**Suppl. Table S6**. **Gene Ontology (GO) enrichment.** *Available as a separate excel worksheet.*

SetRank Gene Ontology (GO) term enrichment analysis performed on candidate loci under selection identified from multivariate GEAs for Acropora muricata and Pocillopora damicornis. Columns show species (A. muricata, P. damicornis), information on significant loci (genomic position, RDA-derived significance values), associated annotated gene information (name, position), associated protein identities, and enriched GO molecular function (MF) terms with corresponding setRank statistics.

**Suppl. Table S7.** **Inbound (ICI) and Outbound (OCI) Connectivity Indices** for *Acropora muricata* and *Pocillopora damicornis* for 15 sampled reefs across the Western Indian Ocean. Inbound and Outbound Connectivity indices indicate the respective area (in km^2^) of reefs either up or down-current of a target reef within a sea distance corresponding to genetic connectivity thresholds. Species-specific F_ST_ value thresholds were set as the average of the regional maximum F_ST_ values to capture average genetic differentiation for reef regions, with *A. muricata:* F_ST_≤0.014 and *P damicornis:* F_ST_≤0.067

See **Supp Fig. S6** for spatial distributions across the Western Indian Ocean.

**Region abbreviations**: SEY = Seychelles, MAU = Mauritius, ROD = Rodrigues.

|  | | | ***A. muricata*** | | ***P. damicornis*** | |
| --- | --- | --- | --- | --- | --- | --- |
| **Code** | **Region** | **Reef Name** | **ICI** | **OCI** | **ICI** | **OCI** |
| **S05** | SEY | Cousine | 6.7 | 27.6 | 42.3 | 27.6 |
| **S06** | SEY | Curieuse | 22.4 | 20.8 | 48.7 | 27.6 |
| **S07** | SEY | Coco | 45.4 | 8.0 | – | – |
| **S03** | SEY | Ile Au Cerf | 20.5 | 28.9 | 20.5 | 52.4 |
| **S04** | SEY | Baie Ternay | 1.9 | 46.2 | 4.6 | 52.4 |
| **S09** | SEY | Anse La Mouche | 12.7 | 37.2 | 18.3 | 52.4 |
| **S12** | MAU | Ile Plate | 68.5 | 46.8 | 217.3 | 124.9 |
| **S01** | MAU | St Felix | 71.2 | 71.1 | 90.5 | 270.2 |
| **S11** | MAU | Balaclava | 25.9 | 63.2 | 235.9 | 102.6 |
| **S13** | MAU | Flic Flac | 73.6 | 71.2 | 287.3 | 139.4 |
| **S02** | MAU | Belle Mare | 152.8 | 81.7 | 221.1 | 104.1 |
| **S10** | MAU | Gd Bourg Melville | 179.6 | 46.8 | 224.1 | 124.9 |
| **S14** | ROD | SEMPA | 8.4 | 150.0 | 52.9 | 150.0 |
| **S15** | ROD | Gd Bassin Bretagne | 126.1 | 73.2 | 138.2 | 141.6 |
| **S16** | ROD | 80 Brisants | 110.2 | 24.0 | 150.0 | 71.7 |
